# Associations of weekly working hours with biological aging and all-cause and cardiovascular mortality: the role of lifestyle

**DOI:** 10.1186/s12995-026-00518-4

**Published:** 2026-06-30

**Authors:** Tiina Föhr, Anna Kankaanpää, Aino Heikkinen, Mikaela Hukkanen, Christer Hublin, Jaakko Kaprio, Miina Ollikainen, Elina Sillanpää

**Affiliations:** 1 Gerontology Research Center (GEREC), Faculty of Sport and Health Sciences, University of Jyväskylä, PO Box 35 (VIV), Jyväskylä, FIN-40014 Finland; 2 Institute for Molecular Medicine Finland (FIMM), HiLife, University of Helsinki, Helsinki, Finland; 3 Minerva Foundation Institute for Medical Research, Helsinki, Finland; 4 Finnish Institute of Occupational Health, Helsinki, Finland; 5 Faculty of Sport and Health Sciences, University of Jyväskylä, Jyväskylä, Finland; 6 Wellbeing Services County of Central Finland, Jyväskylä, Finland

**Keywords:** Biological aging, Lifestyle, Mortality, Working hours, Twin study

## Abstract

**Background:**

This explorative study investigated the associations between working hours, biological aging, and all-cause and cardiovascular disease (CVD) mortality, and assessed the potential mediating role of lifestyle factors.

**Methods:**

We utilized data from the Older Finnish Twin Cohort, which comprised 9246 participants who had reported their weekly working hours in 1990. Biological aging was assessed using blood-based epigenetic aging measures in a subsample (*n* = 740). We used a counterfactual mediation approach. Lifestyle-related factors, including leisure-time physical activity (LTPA), alcohol consumption, body mass index (BMI), smoking, and sleep, were considered potential mediators. Between-within twin models were used to evaluate the extent to which shared familial factors confounded the observed associations.

**Results:**

During the mean 28-year follow-up period, 20.5% of the participants died. Long hours were directly associated with decreased risk of all-cause mortality, but only at the between-pair level; there were no associations with biological aging. However, long hours were indirectly associated with an increased risk of all-cause and CVD mortality through a higher BMI and smoking, and with an increased risk of CVD mortality through lower LTPA levels. Long hours were also indirectly associated with accelerated biological aging through smoking. Part-time work was indirectly associated with a lower risk of all-cause and CVD mortality through a lower BMI.

**Conclusions:**

The findings of this explorative study suggest that direct associations between long working hours, biological aging, and mortality are shaped by lifestyle-mediated pathways and shared familial factors.

**Supplementary Information:**

The online version contains supplementary material available at https://doi.org/10.1186/s12995-026-00518-4.

## Background

Today’s working-age population is expected to remain in the workforce longer than previous generations, so it is crucial to improve the understanding of the relationships between work-related factors, lifestyle, and health. Previous studies have suggested that long working hours are associated with several adverse health outcomes, including an increased risk of cardiovascular diseases (CVD) [1–5] and mortality due to CVD [4, 6].

The association of long working hours and adverse health outcomes could be explained by insufficient recovery from and psychosocial stress related to work, or simply a lack of time and energy, and reduced opportunities to maintain a healthy lifestyle [7, 8]. For instance, long working hours can cause stress, which contributes to cardiovascular damage and increases the risk of stroke [9]. Additionally, overtime work may be associated with health-adverse lifestyle behaviors, such as smoking, heavy alcohol consumption, and physical inactivity [4], as well as with sleep disorders and short sleep duration [10].

Research on the association between long working hours and biological aging, which can reflect cumulative exposure to risk factors that may later emerge as age-related diseases, is limited. Investigating biological aging in this context is thus valuable because it may indicate work-life-related stress and strain, which can increase the risk of future health issues [11–13].

Genome-wide DNA methylation (DNAm) data can be used to construct composite scores [14], for example, epigenetic clocks, which can be used to estimate the biological age of an individual. Biological age provides a summary of the effects of genetic susceptibility and the cumulative effects of lifestyle and environmental factors on physiological aging over the life course [15, 16]. These estimates, called DNAm age, are considered valid measures of biological aging and predict, for instance, time-to-CVD and time-to-death [17–19]. Previous studies suggest that these age estimates produced by epigenetic clocks may serve as indicators of stress/workload effects on human physiology [11–13]. However, the findings of these studies concerning the relationship between working hours and biological aging have been ambiguous [11–13].

Further research is needed to understand the relationship between long working hours, all-cause and CVD mortality, and the accumulated risk of age-related diseases in terms of biological aging. To our knowledge, no previous study has simultaneously investigated these associations within the same study cohort. The purpose of this study was thus to explore these associations and the potential mediating role of lifestyle factors.

## Methods

### Study design and participants

The study participants were twins from the Older Finnish Twin Cohort (FTC) [20]. This study utilizes the second follow-up survey, which was mailed in 1990 to twins born in 1930–1957. The response rate to this survey was 77% (12,502 participants). The participants who had reported being employed and actively engaged in their working lives and had answered questions about their weekly working hours in 1990 were included in the present study (*n* = 9246, 51.3% women). The participants’ mean age (± standard deviation) in 1990, when the questionnaire data were gathered, was 43.0 ± 7.1 years. Blood samples were taken from a subsample of the participants (*n* = 740) during the 1993–2020 period (mean age 61.0 ± 9.3 years), and blood-based DNAm was used to assess their biological aging.

### Questionnaire data

*Working hours* were reported in the following categories: part-time (< 30 h/week), standard hours (30–39 h/week, reference category), slightly long hours (40–44 h/week), long hours (45–54 h/week) and very long hours (≥ 55 h/week).

The volume of *leisure-time physical activity* (LTPA) in metabolic equivalent units (MET index) was based on a series of structured questions on LTPA (monthly frequency, mean duration, and mean intensity of sessions) and physical activity during journeys to and from work. The index was calculated by assigning a multiple of the individual’s resting metabolic rate (MET score) to each activity and by calculating the product of the activity, which was defined as intensity × duration × frequency [21, 22]. The MET index was expressed as the sum score of leisure MET h/day.

*Body mass index* (BMI, kg/m^2^) was calculated based on self-reported weight and height.

The participants’ *smoking status* was categorized as never smoked, occasional smoker, former smoker, current light smoker (1–9 cigarettes per day [CPD]), medium smoker (10–19 CPD), and heavy smoker (≥ 20 CPD) based on the participants’ responses to a detailed smoking behavior and history questionnaire [23].

*Alcohol consumption* was measured via the consumption frequency and quantity of specific types of beverages and converted into the number of grams of absolute ethanol per day. The participants were categorized as never, former, occasional (> 0.1 and < 1.3 g), low (≥ 1.3 and < 25 g), medium (≥ 25 and < 45 g), high (≥ 45 and < 65 g), and very high alcohol consumers (≥ 65 g) [24].

Information on *sleep length* was obtained by asking the participants how many hours they usually slept per 24 h. Nine response alternatives were used (≤ 6, 6.5, 7, 7.5, 8, 8.5, 9, 9.5, and ≥ 10 h). Sleep length was further categorized into three classes: short (< 7 h), average (7–8 h), and long (> 8 h) [25]. Information on *sleep quality* was obtained by asking how often they suffered from insomnia. The response alternatives were ‘every night or almost every night’, ‘3–5 nights a week’, ‘1–2 nights a week’, ‘less than 1 night a week’ and ‘never or more seldom than once a month’ and was further categorized as poor (‘every night or almost every night’ or ‘3–5 nights a week’), fairly poor (‘1–2 nights a week’) and good (‘less than 1 night a week’ or ‘never or more seldom than once a month’) [26].

### Confounding variables

Information on *work schedules* was obtained by assessing each participant’s work type, which was classified into five categories: fixed days, fixed nights, rotating shift with a two-shift pattern without a night shift, rotating shift with a two-shift pattern with a night shift, and rotating shift with a three-shift pattern. We further combined these categories as follows: fixed days, fixed nights, shift work without night work, and shift work with night work [27].

Three *socioeconomic status* (SES) categories (white-, grey-, and blue-collar) were defined by years of education and the amount of physical activity at work [28].

### Mortality follow-up

The *mortality follow-up* began on the date of the participants’ responses to the 1990 questionnaire. The follow-up continued through 31 December 2020, and CVD and all-cause mortality was analyzed during this period. Mortality data – with exact death dates, causes of death, and emigration from Finland status – were available from Statistics Finland. All-cause mortality included all events leading to death. CVD mortality indicated that the underlying cause of death was classified as ischemic heart diseases (International Classification of Diseases [ICD]-10 codes I20–I25), other heart diseases excluding rheumatic and alcohol-related conditions (ICD-10 codes I30–I42.5, I42.7–I52), cerebrovascular diseases (ICD-10 codes I60–I69), hypertension, pulmonary heart disease, diseases of pulmonary circulation, diseases of the arteries, arterioles/capillaries, veins, lymphatic vessels and lymph nodes, as well as other unspecified disorders of the circulatory system (ICD-10 codes I10–I15, I26–I28, I70–I99) [29, 30]. Equivalent ICD-9 codes were used for the period between 1990 and 1995.

### Biological aging

*Biological aging* was assessed in a subsample of the participants using blood-based epigenetic aging measures: principal component (PC)-based DNAm GrimAge [19, 31] and DunedinPACE [18]. DNAm levels were determined using Illumina’s Infinium HumanMethylation450 BeadChips (450k, *n* = 279) or the Infinium MethylationEPIC BeadChips (EPIC version 1, *n* = 338, and EPIC version 2, *n* = 123). The measurement and preprocessing of the DNAm levels as well as additional information on epigenetic aging measures have recently been described in detail [32]. To summarize, DNAm GrimAge, which is a composite of age, sex, DNAm-based surrogates for seven plasma proteins and smoking pack-years, was designed to predict mortality [19]. Further, PC-based epigenetic clocks have recently been developed to bolster the reliability and validity of epigenetic clocks [31]. We produced PC-based GrimAge estimates using an R package (https://github.com/MorganLevineLab/PC-Clocks). DunedinPACE was trained to predict the pace of aging, which describes changes in biomarkers of organ–system integrity over 20 years [18]. Estimates for the pace of aging (in years/calendar year) were produced using an R package (https://github.com/danbelsky/DunedinPACE). Age acceleration (AAPC-Grim) in years was calculated as the residual from a regression of epigenetic age estimates on chronological age, the measurement platform (BeadChip) and their interaction. The estimates of pace of aging were residualized for the same factors before further analysis.

### Statistical analysis

**Fig. 1:**
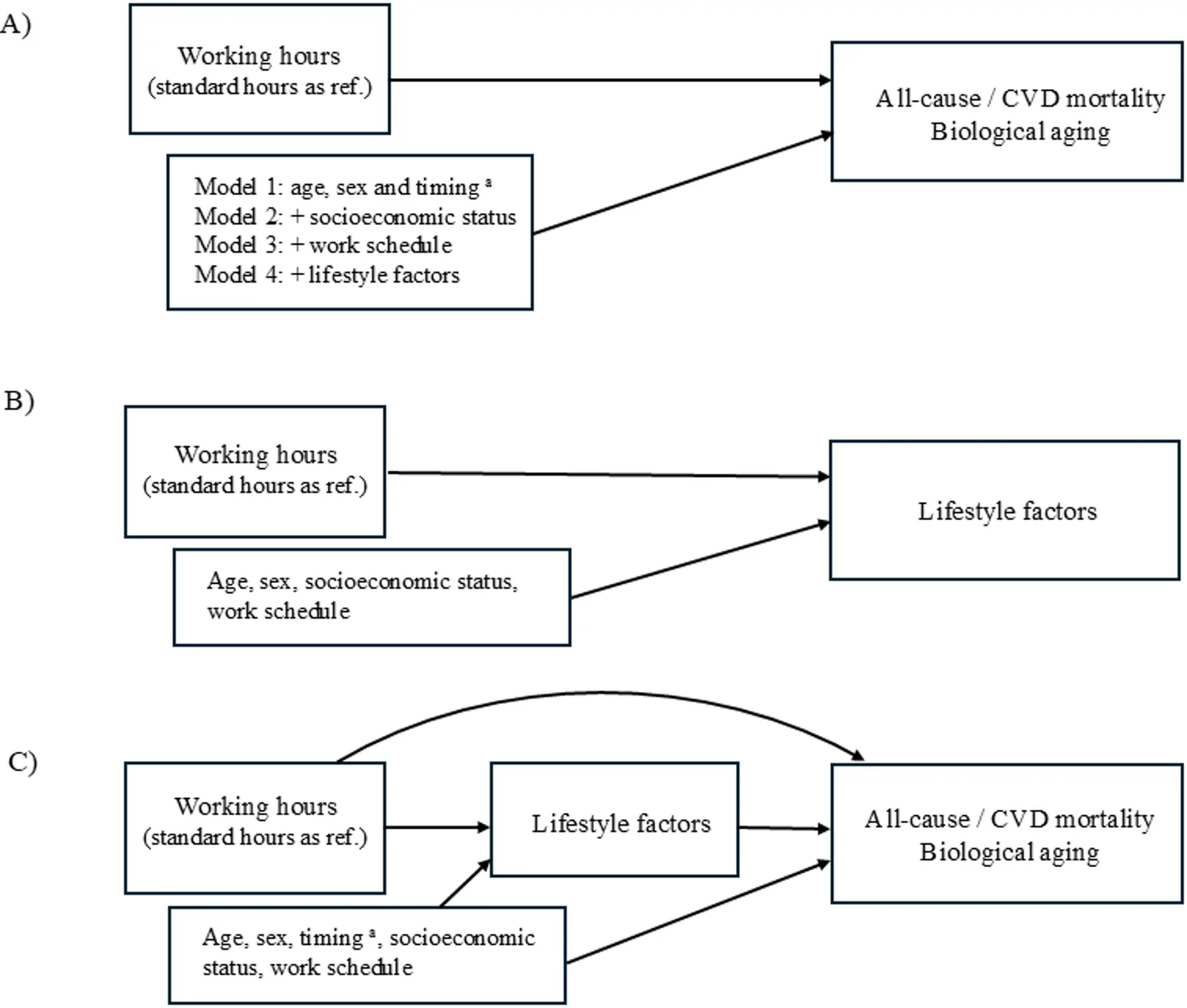
Overview of the estimated models **A**) Overall model assessing the associations between working hours and mortality or biological aging, **B**) Model for associations between working hours and lifestyle factors, and **C**) Model for estimating indirect associations through each lifestyle factor one at a time and through multiple lifestyle factors simultaneously. CVD, cardiovascular disease; Ref., reference category. ^a^ Timing of the blood drawn was adjusted for only in the models of biological aging

The analyses were conducted using R (version 4.5.1). An overview of the estimated models is presented in Fig. 1.

First, we assessed the overall association of weekly working hours with all-cause and CVD mortality using survival models. We applied both Cox proportional hazards model and Aalen’s additive hazards model, as the latter was more suitable for the subsequent analysis of indirect associations within counterfactual mediation framework [33, 34]. The associations were adjusted for baseline age and sex in Model 1, baseline age, sex, and SES in Model 2, baseline age, sex, SES, and work schedule in Model 3, and baseline age, sex, SES, work schedule, and lifestyle factors including LTPA, BMI, alcohol consumption, smoking, sleep length, and sleep quality in Model 4. We also examined the association of weekly working hours with biological aging using linear regression models, which were adjusted in the same way as the survival models and additionally for timing of the blood drawn.

Second, we decomposed the total association of weekly working hours on mortality into the indirect and direct associations within counterfactual mediation framework. The natural indirect effect (NIE) represents the association of exposure on the outcome through the lifestyle factor, while the natural direct effect (NDE) indicates the association of exposure on the outcome not explained by the lifestyle factor.

In our survival analysis, the NIEs and NDEs presented on the hazard difference scale (per 100,000 person-years) were estimated counterfactually by changing the weekly working hours category from standard hours (reference) to part-time, slightly long, long, and very long hours. We initially examined the NIEs through one lifestyle factor at a time using an inverse weighting approach [33]. The weights were calculated based on the expected probabilities from the estimated linear and multinomial regression models between the working hours and lifestyle factors. We then assessed the NIEs simultaneously through multiple pathways [34]. Details of the modelling procedure are presented in the Supplementary Text.

We also estimated the NIEs and NDEs for biological aging. The procedure was similar to that used for survival outcomes, except that Aalen’s model was replaced with a linear regression model. The NIEs and NDEs were presented as differences on the original scale of the outcomes.

All survival and related models examining indirect associations for one lifestyle factor at a time, were also estimated stratified by sex. Finally, we utilized a twin study design and applied a between-within modeling framework. The overall associations of working hours with lifestyle factors, biological aging, and mortality were decomposed into between-pair and within-pair effects to assess confounding by shared familial influences including genetic and early environmental influences. Between-pair effects capture family-level associations whereas within-pair effects reflect associations independent of shared familial influences. Details of the modelling procedure are presented in the Supplementary Text.

## Results

### Participants’ characteristics

The baseline characteristics of all the participants and the subsample with DNAm data are presented separately in Table 1. Most of the participants worked standard hours (41.8%). The men tended to work long hours more frequently than the women (Table S1).

**Table 1:** Characteristics of the participants

	All (*N* = 9246)	Missing cases *N*	Subsample (*N* = 740)	Missing cases *N*
Sex, N (%) of participants				
Female	4740 (51.3)		530 (71.6)	
Male	4506 (48.7)		210 (28.4)	
Age in 1990, mean (SD) range, years	43.0 (7.1) 33–60		45.1 (7.9) 33–60	
Age at subproject (blood sample collection for DNAm data), mean (SD) range, years			61.0 (9.3) 37–81.5	
DNAm age, mean (SD)				
DNAm PC-GrimAge			69.5 (7.8)	
Age acceleration (AAPC-Grim)			0.00 (4.00)	
Pace of aging (DunedinPACE), in years/calender year			0.98 (0.12)	
Physical activity, mean (SD) METh/day	3.4 (3.4)	83	3.1 (2.7)	
Body mass index, mean (SD), kg/m^2^	24.4 (3.5)	53	24.3 (3.8)	4
Cigarette smoking, N (%) of participants		232		11
Never	4164 (46.2)		405 (55.6)	
Occasional	314 (3.5)		26 (3.6)	
Former	2108 (23.4)		150 (20.6)	
Light (1–9 cigarettes/day)	604 (6.7)		45 (6.2)	
Medium (10–19 cigarettes/day)	973 (10.8)		60 (8.2)	
Heavy (20 + cigarettes/day)	851 (9.4)		43 (5.9)	
Alcohol consumption, N (%) of participants		75		2
Lifetime abstainer	932 (10.2)		100 (13.6)	
Former	75 (0.8)		4 (0.5)	
Occasional	418 (4.6)		40 (5.4)	
Low	6833 (74.5)		547 (74.1)	
Medium	580 (6.3)		30 (4.1)	
High	221 (2.4)		12 (1.6)	
Very high	112 (1.2)		5 (0.7)	
Sleep length		32		3
< 7 h	1723 (18.7)		149 (20.2)	
7–8 h	6430 (69.8)		516 (70.0)	
> 8 h	1061 (11.5)		72 (9.8)	
Sleep quality		16		1
Good	7656 (82.9)		612 (81.7)	
Fairly poor	1077 (11.7)		76 (10.1)	
Poor	497 (5.4)		61 (8.1)	
Weekly working hours, N (%)				
Part-time (< 30 h/week)	520 (5.6)		54 (7.3)	
Standard hours (30–39 h/week)	3862 (41.8)		368 (49.7)	
Slightly long hours (40–44 h/week)	3683 (39.8)		243 (32.8)	
Long hours (45–54 h/week)	773 (8.4)		53 (7.2)	
Very long hours (≥ 55 h/week)	408 (4.4)		22 (3.0)	
Work schedule, N (%)		73		1
Day	7453 (81.2)		601 (81.3)	
Night	106 (1.2)		8 (1.1)	
Shift without night	880 (9.6)		76 (10.3)	
Shift with night	734 (8.0)		54 (7.3)	
Socioeconomic status, N (%)		229		20
Blue-collar	3549 (39.4)		255 (35.4)	
Grey-collar	4314 (47.8)		333 (46.3)	
White-collar	1154 (12.8)		132 (18.3)	
Type of employment				
Hourly or monthly wage	7775 (84.1)		654 (88.4)	
Piecework	587 (6.3)		25 (3.4)	
Self-employed (other than farmer)	773 (8.4)		52 (7.0)	
Farmer	111(1.2)		9 (1.2)	

DNAm, DNA methylation; MET, metabolic equivalent; SD, standard deviation

Of the 9246 individuals in this study, 1895 (20.5%, 64.4% men) died during the mean follow-up period of 28.0 years (range 0.4–30.1). Among these deaths, 517 (27.3%, 72.9% men) were due to CVD.

### Associations of working hours with mortality and biological aging

Overall, a linear association between working hours and all-cause or CVD mortality was not detected, except in the lifestyle-adjusted model for all-cause mortality, where a negative trend was observed (Table 2; Fig. 1A). Long hours was associated with a lower risk of all-cause mortality compared with standard hours. After further adjustments (Model 3), the association was slightly attenuated, but in the lifestyle-adjusted model, the association was even stronger. Weekly working hours were not associated with CVD mortality.

**Table 2:** The association of weekly working hours with mortality outcomes for the entire sample

	Working hours (standard hours as ref.)	Number of deaths / *n*^a^	HD	95% CI ^b^	HR	95% CI ^b^
**All-cause mortality**						
Model 1		1,895 / 9,246				
	Part-time	104 / 520	2	-130, 134	1.047	0.854, 1.284
	Standard hours	740 / 3,862	0	-	1.000	-
	Slightly long hours	806 / 3,683	-3	-70, 64	1.012	0.913, 1.121
	Long hours	**147 / 773**	**-177**	**-293**,** -61**	**0.802**	**0.670**,** 0.959**
	Very long hours	98 / 408	23	-137, 184	1.086	0.874, 1.350
				*p* = 0.207		*p* = 0.413
Model 2		1,841 / 9,017				
	Part-time	100 / 506	7	-126, 141	1.063	0.863, 1.309
	Standard hours	725 / 3,786	0	-	1.000	-
	Slightly long hours	776 / 3,564	-13	-83, 57	0.985	0.887, 1.095
	Long hours	**145 / 759**	**-160**	**-276**,** -45**	**0.821**	**0.685**,** 0.984**
	Very long hours	95 / 402	2	-161, 165	1.048	0.842, 1.304
				*p* = 0.159		*p* = 0.280
Model 3		1,824 / 8,945				
	Part-time	96 / 495	-13	-145, 119	1.030	0.830, 1.279
	Standard hours	720 / 3,763	0	-	1.000	-
	Slightly long hours	772 / 3,546	-9	-80, 63	0.992	0.891, 1.104
	Long hours	**145 / 748**	**-151**	**-271**,** -30**	0.835	0.696, 1.003
	Very long hours	91 / 393	-5	-170, 160	1.046	0.835, 1.311
				*p* = 0.232		*p* = 0.398
		/ 8,554				
Model 4	Part-time	92 / 478	30	-110, 170	1.104	0.881, 1.384
	Standard hours	678 / 3,620	0	-	1.000	-
	Slightly long hours	715 / 3,367	-36	-106, 34	0.959	0.857, 1.072
	Long hours	**137 / 719**	**-215**	**-331**,** -100**	**0.791**	**0.657**,** 0.953**
	Very long hours	83 / 370	-109	-282, 64	0.893	0.695, 1.148
				*p* = 0.004		*p* = 0.842
**CVD mortality**						
Model 1		517 / 9,242				
	Part-time	24 / 520	-10	-76, 57	1.018	0.666, 1.556
	Standard hours	181 / 3,861	0	-	1.000	-
	Slightly long hours	225 / 3,680	10	-25, 45	1.098	0.901, 1.338
	Long hours	56 / 773	16	-53, 86	1.161	0.854, 1.579
	Very long hours	31 / 408	40	-51, 131	1.333	0.899, 1.977
				*p* = 0.294		*p* = 0.131
Model 2		501 / 9,013				
	Part-time	24 / 506	-2	-69, 66	1.084	0.711, 1.652
	Standard hours	178 / 3,785	0	-	1.000	-
	Slightly long hours	215 / 3,561	4	-32, 39	1.039	0.849, 1.271
	Long hours	55 / 759	19	-52, 89	1.171	0.858, 1.599
	Very long hours	29 / 402	22	-67, 111	1.215	0.808, 1.826
Model 3		499 / 8,941				
	Part-time	24 / 495	-1	-71, 69	1.096	0.712, 1.688
	Standard hours	178 / 3,762	0	-	1.000	-
	Slightly long hours	214 / 3,543	5	-31, 40	1.045	0.852, 1.282
	Long hours	55 / 748	22	-48, 92	1.194	0.872, 1.634
	Very long hours	28 / 393	21	-73, 115	1.225	0.807, 1.859
				*p* = 0.519		*p* = 0.323
Model 4		477 / 8,554				
	Part-time	24 / 478	21	-53, 96	1.270	0.818, 1.974
	Standard hours	169 / 3,619	0	-	1.000	-
	Slightly long hours	204 / 3,364	2	-33, 37	1.031	0.832, 1.277
	Long hours	53 / 719	3	-68, 74	1.144	0.831, 1.574
	Very long hours	27 / 370	-3	-100, 94	1.062	0.682, 1.653
				*p* = 0.842		*p* = 0.833

CI, confidence interval; HD, hazard difference estimated from Aalen model (presented per 100,000 person-years); HR, hazard ratio estimated from Cox model; Ref., reference category. Model 1 was adjusted for baseline age and sex; Model 2 for baseline age, sex and socioeconomic status; Model 3 for baseline age, sex, socioeconomic status and work schedule; and Model 4 for baseline age, sex, socioeconomic status, work schedule and lifestyle factors (leisure-time physical activity, body mass index, alcohol consumption, smoking, sleep length and sleep quality)

*P* values for linear trend

^a^ Four participants had missing data on the cause of death

^b^ CIs were corrected for clustered sampling within families

Statistically significant associations are presented in bold

We found a linear trend between longer hours and decelerated biological aging when using AAPC-Grim. After adjusting for lifestyle factors, the trend was no longer significant (Table 3; Fig. 1A). The participants who worked part-time aged 1.3 years slower as measured by AAPC-Grim, compared with those who worked standard hours. The difference remained significant after further adjustments (Model 4).

**Table 3:** The association of weekly working hours with biological aging as measured by AAPC-Grim and DunedinPACE

Model	Working hours (standard hours as ref.)	AAPC-Grim		DunedinPACE	
		b	95% CI ^a^	b	95% CI ^a^
Model 1	Part-time	**-1.308**	**-2.216**,** -0.399**	-0.027	-0.058, 0.005
	Standard hours	0.000	-	0.000	-
	Slightly long hours	0.413	-0.214, 1.04	0.014	-0.005, 0.034
	Long hours	0.571	-0.481, 1.623	0.009	-0.025, 0.043
	Very long hours	0.878	-0.767, 2.523	0.019	-0.035, 0.073
			*p* = 0.004		*p* = 0.040
Model 2	Part-time	**-1.191**	**-2.13**,** -0.251**	-0.021	-0.053, 0.01
	Standard hours	0.000	-	0.000	-
	Slightly long hours	0.534	-0.115, 1.184	0.013	-0.007, 0.034
	Long hours	0.692	-0.372, 1.755	0.01	-0.024, 0.044
	Very long hours	0.982	-0.649, 2.614	0.019	-0.033, 0.071
			*p* = 0.003		*p* = 0.067
Model 3	Part-time	**-1.200**	**-2.139**,** -0.260**	-0.023	-0.055, 0.009
	Standard hours	0.000	-	0.000	-
	Slightly long hours	0.547	-0.101, 1.195	0.013	-0.007, 0.033
	Long hours	0.740	-0.345, 1.825	0.007	-0.027, 0.041
	Very long hours	0.971	-0.666, 2.609	0.016	-0.036, 0.069
			*p* = 0.002		*p* = 0.089
Model 4	Part-time	**-0.940**	**-1.819**,** -0.061**	-0.014	-0.045, 0.017
	Standard hours	0.000	*-*	0.000	*-*
	Slightly long hours	0.305	-0.243, 0.854	0.008	-0.011, 0.026
	Long hours	0.375	-0.673, 1.423	-0.003	-0.038, 0.032
	Very long hours	0.012	-1.627, 1.651	0.000	-0.056, 0.057
			*p* = 0.069		*p* = 0.491

b, unstandardized regression coefficient; CI, confidence interval; Ref., reference category. Model 1 was adjusted for baseline age, sex, and timing of the blood drawn; Model 2 for baseline age, sex, timing of the blood drawn and socioeconomic status; Model 3 for baseline age, sex, timing of the blood drawn, socioeconomic status and work schedule; and Model 4 for baseline age, sex, timing of the blood drawn, socioeconomic status, work schedule and lifestyle factors (leisure-time physical activity, body mass index, alcohol consumption, smoking, sleep length and sleep quality)

*P* value for linear trend. Statistically significant associations are presented in bold

^a^ CIs were corrected for clustered sampling within families

### Associations of working hours with lifestyle factors

The results of the linear and multinomial regression analyses between working hours and lifestyle factors are presented in Supplementary Tables S2 and S3, respectively (Fig. 1B). Long and very long hours were associated with lower levels of LTPA and higher BMI compared with standard hours, whereas part-time work was associated with a lower BMI (Supplementary Table S2).

Compared with the participants who had never smoked, slightly long or long hours was associated with higher odds of being a current medium smoker, while long hours was associated with higher odds of being a heavy smoker (Supplementary Table S3). In contrast, part-time workers had lower odds of being heavy smokers.

Long hours were associated with higher odds of short sleep (< 7 h), whereas slightly long and very long hours were associated with lower odds of long sleep, compared with sleeping 7–8 h. In contrast, working part-time was associated with higher odds of sleeping 8.5 h or more. Working hours were not consistently associated with alcohol consumption or sleep quality (Supplementary Table S3), and these variables were therefore not considered further as potential mediators.

In the smaller subsample of participants with information on biological aging, the associations of weekly working hours with lifestyle factors did not consistently reach statistical significance (data not shown). However, long and very long hours were associated with higher odds of being a current smoker compared with having never smoked (OR = 2.09, 95% CI [1.08, 4.04]).

### Indirect associations of working hours with all-cause and CVD mortality through lifestyle factors

**Fig. 2:**
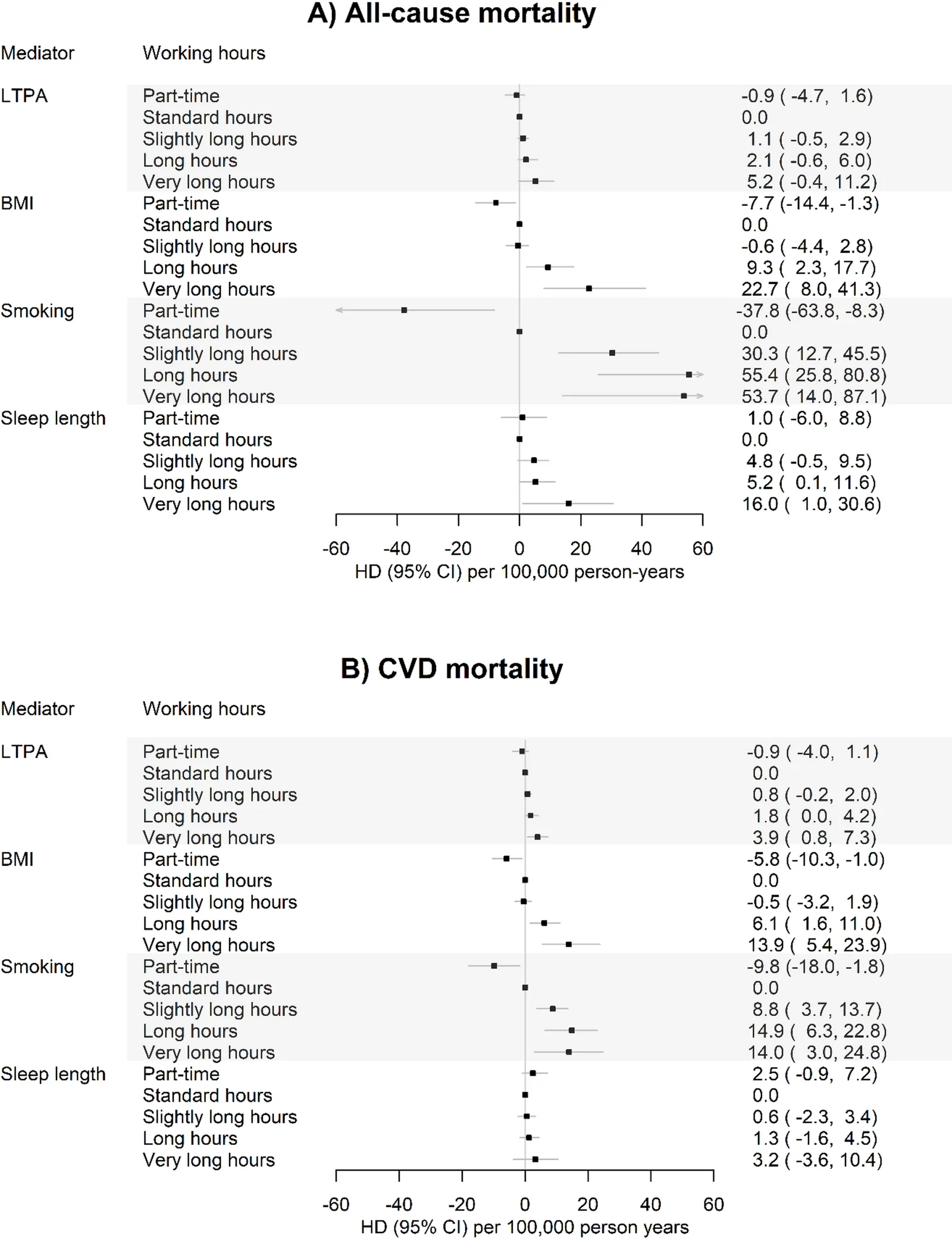
Natural indirect effects of working hours on **A**) all-cause and **B**) cardiovascular disease (CVD) mortality on the hazard difference (HD) scale (per 100,000 person-years) through one mediator at a time (*n* = 8550). BMI, body mass index; LTPA, leisure-time physical activity. Standard working hours was treated as the reference category. LTPA and BMI were modeled as continuous, with smoking (never smoked [reference], occasional, former, light, medium, and heavy smoker) and sleep length (7–8 h [reference], < 7 h and > 8 h) as categorical. The model was controlled for age, sex, socioeconomic status, and work schedule. The confidence intervals were corrected for non-independence due to family relatedness using a bootstrap approach

When indirect associations were estimated via a single lifestyle factor at a time (Figs. 1C and 2A), in comparison to working standard hours, working part-time was associated with fewer all-cause deaths through a lower BMI and fewer all-cause deaths through less frequent smoking. In contrast, long and very long hours were associated with additional deaths through a higher BMI. Slightly long, long, and very long hours were associated with additional deaths through more frequent smoking. Moreover, very long hours was associated with additional deaths through shorter sleep.

Compared to working standard hours, part-time was associated with fewer CVD deaths through a lower BMI and less frequent smoking (Fig. 2B). Very long hours was associated with additional CVD deaths through lower levels of LTPA. Long and very long hours were associated with additional CVD deaths through a higher BMI. Slightly long, long and very long hours were associated with additional CVD deaths through more frequent smoking.

When the indirect associations were estimated simultaneously through multiple routes, the results were very similar (Supplementary Figure S1). The only exception was observed for sleep length; after adjusting for other lifestyle-related factors, working very long hours was no longer indirectly associated with additional deaths through shorter sleep. The NDEs of weekly working hours on all-cause and CVD mortality, which reflected the direct associations after adjusting for lifestyle-related factors, were non-significant. The only exception was the NDE of long working hours on all-cause mortality (Supplementary Table S4).

### Indirect associations of working hours with biological aging through lifestyle factors

The indirect associations of working hours with biological aging were estimated only through smoking, as it was the only lifestyle factor associated with working hours in the smaller subsample. We found that working long or very long hours was indirectly associated with accelerated aging (AAPC-Grim) and a faster pace of aging (DunedinPACE) through more frequent smoking (Supplementary Figure S2, Fig. 1C). The direct association of working part-time on slower aging, as measured by AAPC-Grim, independent of smoking, did not reach significance.

### The sex-specific associations of working hours with all-cause and CVD mortality

For further stratified analyses, the categories of long and very long hours were combined. The total association between long or very long hours and lower all-cause mortality was observed only in men (Supplementary Tables S5). In contrast, long or very long hours were associated with increased CVD mortality in the women (Supplementary Table S5). Long or very long hours were associated with lower levels of LTPA and a higher BMI only in the men (Supplementary Table S6), whereas no clear sex differences were observed in the associations with smoking and sleep length (Supplementary Table S7). Long or very long hours were indirectly associated with an increased risk of mortality through a higher BMI and lower LTPA only in men (see Supplementary Figures S3 and S4).

### The associations of working hours with mortality, biological aging and lifestyles on between- and within-pair level

After decomposing the total associations into between-pair and within-pair components, we observed that long working hours were associated with lower all-cause mortality only at the between-pair level (Supplementary Table S8). In the models for biological aging, the association between part-time work and slower biological aging was likewise observed only at the between-pair level (Supplementary Tables S9). No statistically significant associations were observed at the within-pair level for either outcome (Supplementary Tables S8 and S9). Very long working hours were associated with higher BMI at within-pair level, whereas part-time work was associated with a lower BMI only at between-pair level (Supplementary Tables S10). Long and very long working hours were consistently associated with lower levels of LTPA at both between-pair and within-pair levels, although not all associations reached significance. Long working hours were associated with higher odds of being a heavy smoker at within-pair level (Supplementary Table S11). Very long hours were associated with higher odds of short sleep compared with sleeping 7–8 h at both levels. In contrast, working part-time was associated with higher odds of sleeping 8.5 h or more at within-pair level.

## Discussion

To the best of our knowledge, this is the first study to investigate the role of lifestyle factors as potential mediating pathways in the association of long working hours with biological aging and mortality. Our findings showed that long working hours were not overall associated with accelerated biological aging or an increased risk of mortality. However, long working hours were associated with unhealthier lifestyle factors, predominantly more frequent smoking, a higher BMI, and lower LTPA levels. Furthermore, long working hours were indirectly associated with an increased risk of mortality through these unhealthy lifestyle factors and with accelerated biological aging through smoking. In contrast, long working hours were directly associated with decreased risk of death, suggesting mechanisms not involving traditional risk factors. We further found that part-time work was associated with decelerated biological aging as well as a lower risk of mortality through a lower BMI. These associations were independent of SES and work schedule (i.e., shift work). Our findings complement previous research in the field. For example, recent research has examined the role of lifestyle factors in the association between overtime work and health outcomes, although focusing on self-reported physical and mental health [35].

Previous studies have similarly not supported an association between overtime work and all-cause mortality [6, 36]. On the contrary, our findings indicated that, compared to standard hours, long hours (45–54 h/week) was associated with lower all-cause mortality. However, this association was observed only at the between-twin pair level, suggesting confounding by shared familial factors. The observed association may also indicate selection bias, as particularly at older ages, healthy people are more likely to work longer hours than their less healthy peers. We also examined the causes of death across the working hour categories and found that deaths from neoplasms, as well as accidents and violence (excluding accidental alcohol poisoning), were more prevalent in all the other categories compared with the long working hour (data not shown). This partly explained the observed association with all-cause mortality. It should be noted that after 1990, the severe recession in Finland first led to a major increase in unemployment with job losses associated with excess mortality [37]; perhaps those working long hours were kept employed more often than others. Moreover, long working hours may be more common in high-status occupations (e.g., physicians), which are often associated with better healthcare access and other benefits. It is possible that the SES variable used in our analyses did not fully capture these occupational differences, as it combines education and physical work demands and therefore may not fully account for heterogeneity within occupational groups. In particular, it does not directly correspond to classifications such as white-, blue-, and pink-collar work, and may thus not fully capture differences in work context that could modify the association between long working hours and the outcomes. However, by leveraging the co-twin design and applying within-pair analyses, we were able to partially account for shared familial factors, including genetic influences that are known to be associated with SES [38].

Findings concerning the association of long working hours with CVD mortality have been somewhat ambiguous. For instance, in their large multicohort study in four European countries, Ervasti et al. [6] found that long hours (≥ 50 h/week) was associated with an elevated risk of CVD mortality before the age of 65 but not with maximum follow-up. A large cohort study undertaken by Alicandro et al. [39] did not support an association between long hours and CVD mortality among men, although it did suggest a possible excessive risk among women. This is in line with our findings, which showed that the women who worked long hours had an increased risk of CVD mortality. Descatha et al. [4] concluded in their meta-analysis that existing evidence was inadequate to demonstrate the harmful effects of long working hours on stroke mortality.

Often interrelated unhealthy lifestyles are known to increase the risk of CVD and all-cause mortality [40]. Our findings are in line with those of cross-sectional studies showing that people who work long hours have a higher prevalence of smoking, obesity, physical inactivity [5, 41, 42] and sleep conditions [10]. We found that long (45–54 h/week) and very long (≥ 55 h/week) working hours were indirectly associated with all-cause and CVD mortality through a higher BMI and smoking. In addition, slightly long working hours (40–44 h/week) were indirectly associated with mortality outcomes through smoking. Interestingly, we also found that part-time work (< 30 h/week) was indirectly associated with a lower risk of all-cause and CVD mortality through a lower BMI.

We investigated the association between weekly working hours and biological aging in a smaller subsample of participants. We opted to use second- (GrimAge) and third-generation (DunedinPACE) epigenetic clocks, as previous findings have demonstrated technically more robust and consistent associations in the anticipated directions with social factors compared to first-generation clocks [11, 43–45]. We found part-time work to be overall associated with a slower aging process. Previous studies using second- and third-generation clocks, but varying reference categories for long working hours, have not found an association between long working hours and biological aging [11, 12]. Compared to these previous studies, our study had more specific working hour categories, including a category for part-time work, and a reference group that excluded part-time workers. However, our findings indicated that the association was present only at the between-twin pair level, suggesting confounding by shared familial factors. Smoking is a recognized determinant of accelerated biological aging [44]. Consistent with this, our results showed that long working hours were indirectly associated with accelerated biological aging through smoking.

This study had some limitations. First, working life has changed in many ways since the 1990s, limiting the applicability of our findings to the present day. Second, working hours were categorized based on participants’ responses to a questionnaire with predefined options reflecting the structure of Finnish working life in the 1990s. We thus considered working 30–39 h per week as standard hours. Previous studies have often categorized long working hours into the three analytical categories of 41–48, 49–54, and ≥ 55 h per week and compared these to standard working hours (35–40 h/week). This discrepancy complicates the comparability of our findings with previous studies. Third, in the sex-stratified mortality analyses, the number of women (and their deaths) in the categories of long and very long working hours was small. Fourth, the blood samples were collected over a long period, and our analysis of biological aging does not explicitly account for potential temporal variation in the associations of interest. However, we adjusted our models for the timing of blood collection (i.e., time elapsed from the start of follow-up until blood draw), and the effect of this variable was practically null. Fifth, the number of participants in the working hour categories for the models of biological aging was small, which may have limited statistical power to detect other lifestyle factors than smoking as potential mediating factors. Sixth, the associations between working hours and lifestyle were examined cross-sectionally; thus, causal inferences cannot be made, and reverse causality cannot be ruled out. The aim was to investigate their co-occurrence rather than temporal changes, and changes in working hours or lifestyle factors over time were not captured. In addition, population-level changes in lifestyle behaviors may have influenced the observed associations. For example, since 1990, Finnish men in particular have quit smoking in large numbers [46]. We might speculate that those working long hours quit smoking more often than those working standard hours, leading to a “direct” effect in our models, as changes in lifestyle are not included in the mediator variable set. This would be consistent with the lower risk of deaths from cancer among those working long hours in 1990. Finally, it should be acknowledged that the effect sizes were relatively small.

In addition to the limitations, our study has several strengths. Notably, we were able to distinguish part-time work as a separate category. The findings related to part-time work suggested potential benefits in terms of lower mortality risk, with this association partly explained by a more favorable BMI. These observations are highly relevant in the context of ongoing discussions on reduced working hours, work–life balance, and part-time arrangements. Our results therefore highlight the importance of considering not only the risks of long working hours but also the potential health implications of shorter working time, as well as the role of lifestyle factors in these associations. Finally, a major strength of the study is the twin structure of the data, which allowed us to account for familial factors including shared genetic and environmental influences that are known to influence the associations of interest.

The relationships between working hours, lifestyle factors, and health outcomes such as biological aging and mortality are complex and multidimensional. In addition to working hours, these associations are likely influenced by a range of factors, including family responsibilities, opportunities for recovery and use of leisure time, caregiving roles (e.g., for children or older relatives), and broader social and occupational circumstances. To better understand these complex pathways, further research is needed, for example on the effects of long working hours across diverse occupational groups exposed to varying psychosocial work-related factors, such as differences in the balance between effort and control or rewards. For example, a combination of long working hours and low job control may have a more adverse effect on health behaviors or health than a combination of long working hours and high job control. In addition, future studies should examine changes in working hours over time and their associations with changes in lifestyle behaviors and subsequent health outcomes. Such longitudinal approaches would help to clarify whether increases or decreases in working hours are linked to corresponding changes in health behaviors and, ultimately, to trajectories of biological aging and mortality risk.

## Conclusions

The results of our study support the hypothesis that working life and its demands are associated with employees’ lifestyles. Moreover, the health effects of long working hours may vary depending on employees’ engagement in healthy lifestyles and shared familial factors. Our findings thus provide valuable insights into how working hours can impact lifestyle, premature death, and biological aging.

## Supplementary Information

Below is the link to the electronic supplementary material.

Supplementary Material 1

## Data Availability

The biobanked FTC data can be found within the Biobank of the National Institute for Health and Welfare, Finland (https://thl.fi/en/our-services/thl-biobank1). All biobanked data are publicly available for use by qualified researchers following a standardized application procedure. Because of the consent given by the study participants and the high degree of identifiability of the twin siblings in Finland, the full cohort data cannot be made publicly available. However, the full cohort data are available through the Institute for Molecular Medicine Finland (FIMM) Data Access Committee (DAC) for authorised researchers with an IRB/ethics approval and an institutionally approved study plan. For more details, please contact the FIMM DAC (fimm-dac@ helsinki.fi).

## Abbreviations

AAPC-Grim: Age acceleration based on the principal component DNA methylation GrimAge estimator
BMI: Body mass index
CI: Confidence interval
CVD: Cardiovascular disease
CPD: Cigarettes per day
DNAm: DNA methylation
FTC: Finnish Twin Cohort
HD: Hazard difference
HR: Hazard ratio
ICD: International classification of diseases
LTPA: Leisure-time physical activity
MET: Metabolic equivalent
NDE: Natural direct effect
NIE: Natural indirect effect
OR: Odds ratio
PC: Principal component
SD: Standard deviation
SES: Socioeconomic status
