# Supplementary material for: Associations of weekly working hours with biological aging and all-cause and cardiovascular mortality: the role of lifestyle
Source: J Occup Med Toxicol. 2026 Jun 30;21:29. doi: 10.1186/s12995-026-00518-4 (PMC13591724; doi:10.1186/s12995-026-00518-4)
Supplement: Supplementary file 1 [file 12995_2026_518_MOESM1_ESM.docx]

**Supplementary material**

**Associations of Weekly Working Hours with Biological Aging and All-Cause and Cardiovascular Mortality: The Role of Lifestyle**

**Supplementary Text**

*Statistical analysis*

Each lifestyle factor was first regressed on the exposure (working hours) and potential confounding variables, including baseline age, sex, socioeconomic status (SES), and work schedule. For the continuous variables, which comprised leisure-time physical activity (LTPA) and body mass index) BMI, we used linear regression models, and the standard errors (SEs) were corrected for the cluster sampling within families using the sandwich package in R (1). For the categorical variables (i.e., alcohol use, smoking, and sleep length and quality), we used multinomial logistic regression models (nnet package) with the SEs corrected for clustering (2). If no clear association was observed between weekly working hours and a potential mediator variable, no further analyses were conducted for that variable.

Next, we constructed a new dataset by repeating each observation five times, which corresponded with the five exposure categories. We created a new variable that took the observed exposure value in the first replication and the possible counterfactual exposure values in the subsequent replications. The weights were calculated based on the expected probabilities from the estimated models between the exposure and lifestyle factors. Finally, the hazard differences were estimated using weighted Aalen’s additive hazard’s model (timereg package) (3). The model included both the original and expanded exposure variables as explanatory variables and was adjusted for baseline age, sex, SES, and work schedule. The coefficient (hazard difference) for the original exposure corresponded to the NDE, while that for the expanded exposure corresponded to the NIE.

We estimated percentile-based 95% confidence intervals (CIs) for the NIEs and NDEs using 1000 nonparametric bootstrap samples, which were resampled at the family (cluster) level to account for the dependency structure. For the models that included indirect associations through multiple pathways, the computations were substantially slower. We therefore used 500 bootstrap replicates instead.

We applied a between-within modeling framework to decompose the overall association between working hours and mortality, biological aging and lifestyle factors into between-pair and within-pair components. Before the analysis, dataset was restricted to twin pairs with complete information on working hours for both members of the pair. Between-pair variables were calculated as family-level means of the dummy-coded working hours categories. Within-pair variables were calculated as the deviation of each individual’s values from their corresponding family-level means. Between- and within-pair variables were included as explanatory variables in Cox proportional hazards with cluster-robust standard errors (survival package), linear mixed-effect regression (lme4 package) and multinomial mixed-effects models (mclogit package). Their associations with the outcomes were estimated as fixed effects, while the family identifier was included as a random intercept in the mixed models. All models were adjusted for baseline age and sex.

*Supplementary References:*

1. Zeileis A, Köll S, Graham N. Various versatile variances: An object-oriented implementation of clustered covariances in r. J Stat Softw. 2020;95:1–36.
2. Davenport C, Soule SA, Armstrong DA. Protesting while black? the differential policing of American activism, 1960 to 1990. Am Sociol Rev. 2011 Feb;76(1):152–78.
3. Martinussen T, Scheike TH. Dynamic regression models for survival data. New York: Springer; 2006.

| **Supplementary Table S1** Characteristics of the participants stratified by sex. | | | | |
| --- | --- | --- | --- | --- |
|  | Women  (N=4740) |  | Men  (N=4506) |  |
| Age in 1990, mean (SD) range, years | 42.8 (7.1) 33–60 |  | 43.2 (7.1) 33–60 |  |
| Physical activity, mean (SD) METh/day | 3.4 (3.2) |  | 3.3 (3.6) |  |
| Body mass index, mean (SD), kg/m^2^ | 23.6 (3.7) |  | 25.3 (3.2) |  |
| Cigarette smoking, N (%) of participants |  |  |  |  |
| Never | 2596 (56.1) |  | 1568 (35.8) |  |
| Occasional | 137 (3.0) |  | 177 (4.0) |  |
| Former | 832 (18.0) |  | 1276 (29.1) |  |
| Light (1–9 cigarettes/day) | 390 (8.4) |  | 214 (4.9) |  |
| Medium (10–19 cigarettes/day) | 468 (10.1) |  | 505 (11.5) |  |
| Heavy (20+ cigarettes/day) | 208 (4.5) |  | 643 (14.7) |  |
| Alcohol consumption, N (%) of participants |  |  |  |  |
| Lifetime abstainer | 661 (14.1) |  | 271 (6.0) |  |
| Former | 36 (0.8) |  | 39 (0.9) |  |
| Occasional | 342 (7.3) |  | 76 (1.7) |  |
| Low | 3500 (74.6) |  | 3333 (74.4) |  |
| Medium | 110 (2.3) |  | 470 (10.5) |  |
| High | 26 (0.6) |  | 195 (4.4) |  |
| Very high | 14 (0.3) |  | 98 (2.2) |  |
| Sleep length |  |  |  |  |
| < 7 hours | 799 (16.9) |  | 924 (20.6) |  |
| 7–8 hours | 3286 (69.6) |  | 3144 (70.1) |  |
| > 8 hours | 641 (13.5) |  | 420 (9.3) |  |
| Sleep quality |  |  |  |  |
| Good | 3754 (83.5) |  | 3902 (82.5) |  |
| Fairly poor | 229 (5.1) |  | 268 (5.7) |  |
| Poor | 515 (11.4) |  | 562 (11.9) |  |
| Weekly working hours, N (%) |  |  |  |  |
| Part-time (< 30 h/week) | 399 (8.4) |  | 121 (2.7) |  |
| Standard hours (30–39 h/week) | 2484 (52.4) |  | 1378 (30.6) |  |
| Slightly long hours (40–44 h/week) | 1573 (33.2) |  | 2110 (46.8) |  |
| Long hours (45–54 h/week) | 193 (4.1) |  | 580 (12.9) |  |
| Very long hours (≥ 55 h/week) | 91 (1.9) |  | 317 (7.0) |  |
| Work schedule, N (%) |  |  |  |  |
| Day | 3739 (79.6) |  | 3714 (83.0) |  |
| Night | 69 (1.4) |  | 41 (0.9) |  |
| Shift without night | 558 (11.9) |  | 322 (7.2) |  |
| Shift with night | 338 (7.2) |  | 396 (8.8) |  |
| Socioeconomic status, N (%) |  |  |  |  |
| Blue-collar | 1682 (36.5) |  | 1867 (42.3) |  |
| Grey-collar | 2311 (50.2) |  | 2003 (45.4) |  |
| White-collar | 612 (13.3) |  | 542 (12.3) |  |
| Type of employment |  |  |  |  |
| Hourly or monthly wage | 4254 (89.7) |  | 3521 (78.1) |  |
| Piecework | 244 (5.1) |  | 343 (7.6) |  |
| Self-employed (other than farmer) | 236 (5.0) |  | 537 (11.9) |  |
| Farmer | 6 (0.1) |  | 105 (2.3) |  |
| MET, metabolic equivalent; SD, standard deviation | | | | |

**Supplementary Table 2** The associations of weekly working hours with the continuous lifestyle factors for the entire sample. The results of the linear regression analyses.

| **Working hours (standard hours as ref.)** | **Lifestyle factor** | ***b*** | ***95% CI*** ^a^ |
| --- | --- | --- | --- |
|  | **LTPA** |  |  |
| Part-time |  | 0.15 | -0.20, 0.50 |
| Standard hours |  | 0.00 | - |
| Slightly long hours |  | -0.12 | -0.29, 0.04 |
| Long hours |  | **-0.31** | **-0.60, -0.01** |
| Very long hours |  | **-0.70** | **-1.05, -0.34** |
|  | **BMI** |  |  |
| Part-time |  | **-0.41** | **-0.72, -0.09** |
| Standard hours |  | 0.00 | - |
| Slightly long hours |  | -0.04 | -0.20, 0.13 |
| Long hours |  | **0.37** | **0.11, 0.62** |
| Very long hours |  | **0.78** | **0.37, 1.19** |

b, unstandardized regression coefficient; BMI, body mass index; CI, confidence interval; LTPA, leisure-time physical activity; Ref., reference category.

The model was adjusted for baseline age, sex, socioeconomic status, and work schedule.

^a^ CIs were corrected for clustered sampling within families.

Statistically significant associations are presented in bold.

**Supplementary Table S3** The associations of weekly working hours with categorical lifestyle factors for the entire sample. The results of the multinomial logistic regression analyses.

| **Working hours (standard hours as ref.)** | **Lifestyle factor** | ***OR*** | ***95% CI*** |
| --- | --- | --- | --- |
|  | **Alcohol use** |  |  |
| Part-time | Never vs. low | 1.30 | 0.96, 1.76 |
| Standard hours |  | 1.00 | - |
| Slightly long hours |  | 0.89 | 0.75, 1.06 |
| Long Hours |  | 0.95 | 0.69, 1.31 |
| Very long hours |  | 1.04 | 0.68, 1.59 |
|  | Former vs. low |  |  |
| Part-time |  | 0.45 | 0.11, 1.83 |
| Standard hours |  | 1.00 | - |
| Slightly long hours |  | 0.86 | 0.49, 1.51 |
| Long Hours |  | 0.58 | 0.19, 1.75 |
| Very long hours |  | 1.78 | 0.71, 4.47 |
|  | Occasional vs. low |  |  |
| Part-time |  | **1.64** | **1.13, 2.36** |
| Standard hours |  | 1.00 | - |
| Slightly long hours |  | 1.05 | 0.83, 1.34 |
| Long Hours |  | 1.11 | 0.68, 1.83 |
| Very long hours |  | 1.28 | 0.69, 2.39 |
|  | Medium vs. low |  |  |
| Part-time |  | 0.77 | 0.45, 1.33 |
| Standard hours |  | 1.00 | - |
| Slightly long hours |  | 1.09 | 0.88, 1.34 |
| Long Hours |  | 1.14 | 0.84, 1.54 |
| Very long hours |  | 1.37 | 0.93, 2.00 |
|  | High vs. low |  |  |
| Part-time |  | 1.63 | 0.84, 3.18 |
| Standard hours |  | 1.00 | - |
| Slightly long hours |  | 0.96 | 0.69, 1.34 |
| Long Hours |  | 1.17 | 0.72, 1.88 |
| Very long hours |  | 1.08 | 0.57, 2.04 |
|  | Very high vs. low |  |  |
| Part-time |  | 0.77 | 0.23, 2.56 |
| Standard hours |  | 1.00 | - |
| Slightly long hours |  | 0.67 | 0.43, 1.04 |
| Long Hours |  | 0.66 | 0.32, 1.34 |
| Very long hours |  | 1.03 | 0.46, 2.26 |
|  | **Smoking** |  |  |
| Part-time | Occasional vs. never | 0.90 | 0.53, 1.52 |
| Standard hours |  | 1.00 | - |
| Slightly long hours |  | 0.86 | 0.66, 1.13 |
| Long Hours |  | 1.24 | 0.82, 1.87 |
| Very long hours |  | 1.08 | 0.62, 1.89 |
|  | Former vs. never |  |  |
| Part-time |  | 0.84 | 0.65, 1.08 |
| Standard hours |  | 1.00 | - |
| Slightly long hours |  | 1.03 | 0.91, 1.17 |
| Long Hours |  | 1.07 | 0.86, 1.33 |
| Very long hours |  | 0.76 | 0.56, 1.02 |
|  | Current light vs. never |  |  |
| Part-time |  | 0.67 | 0.43, 1.02 |
| Standard hours |  | 1.00 | - |
| Slightly long hours |  | 1.09 | 0.90, 1.34 |
| Long Hours |  | 0.94 | 0.63, 1.39 |
| Very long hours |  | 0.90 | 0.55, 1.47 |
|  | Current medium vs. never |  |  |
| Part-time |  | 0.87 | 0.59, 1.27 |
| Standard hours |  | 1.00 | **-** |
| Slightly long hours |  | **1.54** | **1.30, 1.82** |
| Long Hours |  | **1.46** | **1.08, 1.96** |
| Very long hours |  | 1.26 | 0.86, 1.84 |
|  | Current heavy vs. never |  |  |
| Part-time |  | **0.57** | **0.35, 0.95** |
| Standard hours |  | 1.00 | **-** |
| Slightly long hours |  | **1.21** | **1.00, 1.46** |
| Long Hours |  | **1.74** | **1.31, 2.30** |
| Very long hours |  | **1.67** | **1.18, 2.36** |
|  | **Sleep length** |  |  |
|  | Less than 7h vs. 7-8 hour |  |  |
| Part-time |  | 0.96 | 0.72, 1.28 |
| Standard hours |  | 1.00 | **-** |
| Slightly long hours |  | **1.32** | **1.16, 1.51** |
| Long Hours |  | **1.35** | **1.09, 1.67** |
| Very long hours |  | **2.18** | **1.70, 2.80** |
|  | 8.5 h or more vs. 7-8 hours |  |  |
| Part-time |  | **1.54** | **1.19, 2.00** |
| Standard hours |  | 1.00 | - |
| Slightly long hours |  | **0.78** | **0.66, 0.91** |
| Long Hours |  | 0.91 | 0.69, 1.19 |
| Very long hours |  | **0.51** | **0.32, 0.81** |
|  | **Sleep quality** |  |  |
|  | Poor vs. good |  |  |
| Part-time |  | 1.46 | 0.97, 2.18 |
| Standard hours |  | 1.00 | **-** |
| Slightly long hours |  | **1.39** | **1.12, 1.74** |
| Long Hours |  | 1.01 | 0.65, 1.55 |
| Very long hours |  | 1.27 | 0.77, 2.10 |
|  | Fairly poor vs. good |  |  |
| Part-time |  | 0.84 | 0.60, 1.15 |
| Standard hours |  | 1.00 | - |
| Slightly long hours |  | 1.03 | 0.88, 1.20 |
| Long Hours |  | 0.97 | 0.74, 1.27 |
| Very long hours |  | 1.14 | 0.81, 1.61 |

CI, confidence interval; OR, odds ratio; Ref., reference category. The model was adjusted for baseline age, sex, socioeconomic status, and work schedule.

^a^ CIs were corrected for clustered sampling within families.

Statistically significant associations are presented in bold.

**Supplementary Table S4** Natural direct effects of weekly working hours on all-cause and cardiovascular disease (CVD) mortality on the hazard difference (HD) scale (per 100,000 person-years) estimated using the model including pathways through multiple lifestyle factors.

|  | **Working hours (standard hours as ref.)** | **HD** | ***95% CI*** ^a^ |
| --- | --- | --- | --- |
| **All-cause mortality** |  |  |  |
|  | Part-time | 53 | -112, 202 |
|  | Standard hours | 0 | - |
|  | Slightly long hours | -60 | -134, 6 |
|  | Long hours | **-216** | **-313, -87** |
|  | Very long hours | -104 | -255, 63 |
| **CVD mortality** |  |  |  |
|  | Part-time | 3 | -60, 69 |
|  | Standard hours | 0 | - |
|  | Slightly long hours | -2 | -37, 35 |
|  | Long hours | -3 | -61, 59 |
|  | Very long hours | -2 | -90, 89 |
| CI, confidence interval; HD, hazard difference estimated from Aalen model; Ref., reference category.  Model was adjusted for baseline age, sex, socioeconomic status, and work schedule.  ^a^ CIs were corrected for clustered sampling within families using bootstrap approach.  Statistically significant associations are presented in bold. | | | |

**Supplementary Table S5** The association of weekly working hours with all-cause and cardiovascular disease (CVD) mortality stratified by sex.

|  |  |  | **Men** | | | | |  | **Women** | | | | |
| --- | --- | --- | --- | --- | --- | --- | --- | --- | --- | --- | --- | --- | --- |
|  | Working hours (standard hours as ref.) ^a^ |  | Number of deaths / n ^b^ | HD | 95% CI ^c^ | HR | 95% CI ^c^ |  | Number of deaths / n ^b^ | HD | 95% CI ^c^ | HR | 95% CI ^c^ |
|  |  |  |  |  |  |  |  |  |  |  |  |  |  |
| **All-cause mortality** | | | |  |  |  |  |  |  |  |  |  |  |
|  |  |  | 1,220 / 4,506 |  |  |  |  |  | 675 / 4,740 |  |  |  |  |
| Model 1 | |  |  |  |  |  |  |  |  |  |  |  |  |
|  | Part-time |  | 37 / 121 | 6 | -335, 346 | 1.007 | 0.712, 1.424 |  | 67 / 399 | 29 | -79, 138 | 1.075 | 0.834, 1.384 |
|  | Standard hours |  | 400 / 1,378 | 0 | - | 1.000 | - |  | 340 / 2,484 | 0 | - | 1.000 | - |
|  | Slightly long hours |  | 582 / 2,110 | 8 | -115, 130 | 1.009 | 0.888, 1.147 |  | 224 / 1,571 | 3 | -60, 65 | 1.008 | 0.847, 1.199 |
|  | Long or very long hours |  | 201 / 897 | -126 | -265, 13 | 0.854 | 0.720, 1.014 |  | 44 / 284 | 39 | -87, 165 | 1.105 | 0.807, 1.515 |
| Model 2 | |  |  |  |  |  |  |  |  |  |  |  |  |
|  | Part-time |  | 36 / 119 | -3 | -340, 335 | 1.016 | 0.716, 1.443 |  | 64 / 387 | 32 | -79, 143 | 1.087 | 0.838, 1.409 |
|  | Standard hours |  | 396 / 1,358 | 0 | - | 1.000 | - |  | 329 / 2,428 | 0 | - | 1.000 | - |
|  | Slightly long hours |  | 563 / 2,052 | -42 | -165, 80 | 0.954 | 0.837, 1.088 |  | 213 / 1,512 | 7 | -58, 71 | 1.018 | 0.851, 1.219 |
|  | Long or very long hours |  | 196 / 883 | **-146** | **-285, -7** | **0.839** | **0.706, 0.998** |  | 44 / 278 | 60 | -66, 186 | 1.181 | 0.862, 1.618 |
| Model 3 | |  |  |  |  |  |  |  |  |  |  |  |  |
|  | Part-time |  | 34 / 117 | -70 | -422, 281 | 0.953 | 0.663, 1.371 |  | 62 / 378 | 28 | -90, 145 | 1.074 | 0.821, 1.404 |
|  | Standard hours |  | 394 / 1,352 | 0 | - | 1.000 | - |  | 326 / 2,411 | 0 | - | 1.000 | - |
|  | Slightly long hours |  | 560 / 2,040 | -36 | -160, 89 | 0.961 | 0.840, 1.098 |  | 212 / 1,506 | 9 | -54, 72 | 1.025 | 0.856, 1.228 |
|  | Long or very long hours |  | 193 / 871 | -135 | -280, 11 | 0.849 | 0.711, 1.012 |  | 43 / 270 | 63 | -67, 192 | 1.186 | 0.861, 1.635 |
| Model 4 | |  |  |  |  |  |  |  |  |  |  |  |  |
|  | Part-time |  | 34 / 114 | 0 | -351, 351 | 1.051 | 0.729, 1.515 |  | 58 / 364 | 50 | -57, 157 | 1.160 | 0.869, 1.549 |
|  | Standard hours |  | 375 / 1,303 | 0 | - | 1.000 | - |  | 303 / 2,317 | 0 | - | 1.000 | - |
|  | Slightly long hours |  | 517 / 1,941 | -70 | -196, 56 | 0.911 | 0.792, 1.048 |  | 198 / 1,426 | -1 | -65, 63 | 1.022 | 0.842, 1.234 |
|  | Long or very long hours |  | 178 / 836 | **-237** | **-381, -94** | **0.750** | **0.622, 0.905** |  | 42 / 253 | 27 | -109, 162 | 1.109 | 0.797, 1.543 |
|  | | | | | |  |  |  |  |  |  |  |  |
| **CVD-mortality** | | | | | |  |  |  |  |  |  |  |  |
|  |  |  | 377 / 4,504 |  |  |  |  |  | 140 / 4,738 |  |  |  |  |
| Model 1 | | |  |  |  |  |  |  |  |  |  |  |  |
|  | Part-time |  | 19 / 121 | -17 | -190, 155 | 0.935 | 0.490, 1.785 |  | 14 / 399 | 5 | -48, 58 | 1.066 | 0.603, 1.884 |
|  | Standard hours |  | 116 / 1,377 | 0 | - | 1.000 | - |  | 65 / 2,484 | 0 | - | 1.000 | - |
|  | Slightly long hours |  | 179 / 2,109 | 22 | -45, 90 | 1.083 | 0.861, 1.361 |  | 46 / 1,571 | 4 | -23, 30 | 1.060 | 0.717, 1.567 |
|  | Long or very long hours |  | 72 / 897 | 22 | -57, 101 | 1.089 | 0.809, 1.459 |  | 15 / 284 | 68 | -4, 140 | **1.976** | **1.116, 3.500** |
| Model 2 | |  |  |  |  |  |  |  |  |  |  |  |  |
|  | Part-time |  | 10 / 119 | -15 | -191, 160 | 0.967 | 0.508, 1.840 |  | 14 / 387 | 11 | -44, 66 | 1.151 | 0.653, 2.026 |
|  | Standard hours |  | 116 / 1,357 | 0 | - | 1.000 | - |  | 62 / 2,428 | 0 | - | 1.000 | - |
|  | Slightly long hours |  | 174 / 2,051 | 5 | -64, 73 | 1.016 | 0.805, 1.283 |  | 41 / 1,510 | 1 | -26, 28 | 1.020 | 0.679, 1.532 |
|  | Long or very long hours |  | 69 / 883 | 8 | -69, 84 | 0.979 | 0.693, 1.383 |  | 15 / 278 | **75** | **3, 148** | **2.181** | **1.223, 3.889** |
| Model 3 | |  |  |  |  |  |  |  |  |  |  |  |  |
|  | Part-time |  | 10 / 117 | -20 | -206, 167 | 0.965 | 0.500, 1.863 |  | 14 / 378 | 14 | -43, 70 | 1.178 | 0.660, 2.100 |
|  | Standard hours |  | 116 / 1,351 | 0 | - | 1.000 | - |  | 62 / 2,411 | 0 | - | 1.000 | - |
|  | Slightly long hours |  | 173 / 2,039 | 8 | -61, 76 | 1.026 | 0.810, 1.299 |  | 41 / 1,504 | 1 | -26, 29 | 1.017 | 0.677, 1.533 |
|  | Long or very long hours |  | 68 / 871 | 12 | -65, 90 | 1.055 | 0.779, 1.429 |  | 15 / 270 | **79** | **3, 155** | **2.732** | **1.234, 3.975** |
| Model 4 | |  |  |  |  |  |  |  |  |  |  |  |  |
|  | Part-time |  | 10 / 114 | 28 | -161, 218 | 1.184 | 0.604, 2.320 |  | 14 / 364 | 22 | -36, 80 | 1.399 | 0.766, 2.556 |
|  | Standard hours |  | 110 / 1,302 | 0 | - | 1.000 | - |  | 59 / 2,317 | 0 | **-** | 1.000 | - |
|  | Slightly long hours |  | 164 / 1,940 | 5 | -64, 73 | 0.976 | 0.762, 1.251 |  | 40 / 1,424 | 1 | -26, 27 | 1.059 | 0.696, 1.611 |
|  | Long or very long hours |  | 65 / 836 | -22 | -101, 57 | 0.946 | 0.692, 1.294 |  | 15 / 253 | 76 | -3, 155 | **2.107** | **1.132, 3.924** |
| CI, confidence interval; HD, hazard difference estimated from Aalen model; HR, hazard ratio estimated from Cox model; Ref., reference category.  Model 1 was adjusted for baseline age, Model 2 for baseline age and socioeconomic status; Model 3 for baseline age, socioeconomic status, and work schedule; Model 4 for baseline age, socioeconomic status, work schedule, and lifestyle factors (leisure-time physical activity, body mass index, alcohol consumption, smoking, sleep length, and sleep quality).  ^a^ Categories long hours and very long hours were combined.  ^b^ Four participants (2 men and 2 women) had missing data on cause of death.  ^c^ CIs were corrected for clustered sampling within families.  Statistically significant associations are presented in bold. | | | | | | | | | | | | | |

**Supplementary Table S6** The associations of weekly working hours with the continuous lifestyle factors for the entire sample stratified by sex. The results of the linear regression analyses.

|  |  | **Men** |  | |  | **Women** |  |
| --- | --- | --- | --- | --- | --- | --- | --- |
| **Working hours (standard hours as ref.)** | **Lifestyle factor** | ***b*** | ***95% CI*** ^a^ |  | | ***b*** | ***95% CI*** ^a^ |
|  | **LTPA** |  |  | |  |  |  |
| Part-time |  | 0.96 | -0.12, 2.03 | |  | -0.03 | -0.34, 0.27 |
| Standard hours |  | 0.00 | - | |  | 0.00 | - |
| Slightly long hours |  | -0.26 | -0.52, 0.01 | |  | -0.01 | -0.22, 0.20 |
| Long or very long hours |  | **-0.63** | **-0.94, -0.32** | |  | -0.03 | -0.50, 0.44 |
|  | **BMI** |  |  | |  |  |  |
| Part-time |  | **-0.66** | **-1.22, -0.10** | |  | **-0.38** | **-0.75, 0.00** |
| Standard hours |  | 0.00 | - | |  | 0.00 | - |
| Slightly long hours |  | 0.05 | -0.18, 0.27 | |  | -0.07 | -0.31, 0.16 |
| Long or very hours |  | **0.67** | **0.40, 0.94** | |  | 0.06 | -0.45, 0.56 |
| b, unstandardized regression coefficient; BMI, body mass index; CI, confidence interval; LTPA, leisure-time physical activity; Ref., reference category.  The model was adjusted for baseline age, sex, socioeconomic status, and work schedule.  ^a^ CIs were corrected for clustered sampling within families.  Statistically significant associations are presented in bold. | | | | | | | |

**Supplementary Table S7** The associations of weekly working hours with the categorical lifestyle factors for the entire sample stratified by sex. The results of the multinomial logistic regression analyses.

|  |  | **Men** |  |  | **Women** | |
| --- | --- | --- | --- | --- | --- | --- |
| **Working hours (standard hours as ref.)** ^a^ | **Lifestyle factor** | ***OR*** | ***95% CI*** ^b^ |  | ***OR*** | ***95% CI*** ^b^ |
|  | **Alcohol use** ^c^ |  |  |  |  |  |
|  | Never vs. low | |  |  |  |  |
| Part-time |  | 1.32 | 0.63, 2.76 |  | 1.31 | 0.94, 1.82 |
| Standard hours |  | 1.00 | - |  | 1.00 | - |
| Slightly long hours |  | 0.74 | 0.54, 1.01 |  | 0.96 | 0.78, 1.18 |
| Long or very long hours | | 0.98 | 0.67, 1.45 |  | 0.86 | 0.57, 1.32 |
|  | Former vs. low | |  |  |  |  |
| Part-time |  | 0.64 | 0.11, 3.67 |  | 0.39 | 0.05, 3.4 |
| Standard hours |  | 1.00 | - |  | 1.00 | - |
| Slightly long hours |  | 0.56 | 0.26, 1.20 |  | 1.30 | 0.61, 2.77 |
| Long or very long hours | | 0.69 | 0.27, 1.76 |  | 1.78 | 0.50, 6.42 |
|  | Occasional vs. low | |  |  |  |  |
| Part-time |  | **3.34** | **1.18, 9.46** |  | **1.49** | **1.01, 2.21** |
| Standard hours |  | 1.00 | - |  | 1.00 | - |
| Slightly long hours |  | 1.15 | 0.62, 2.10 |  | 1.06 | 0.82, 1.38 |
| Long or very long hours | | 1.65 | 0.81, 3.36 |  | 0.97 | 0.55, 1.70 |
|  | Medium vs. low | |  |  |  |  |
| Part-time |  | 0.92 | 0.46, 1.84 |  | 0.56 | 0.22, 1.42 |
| Standard hours |  | 1.00 | - |  | 1.00 | - |
| Slightly long hours |  | 1.03 | 0.81, 1.31 |  | 1.27 | 0.80, 2.00 |
| Long or very long hours | | 1.18 | 0.90, 1.56 |  | 1.39 | 0.64, 3.00 |
|  | High or very high vs. low | | |  |  |  |
| Part-time |  | 1.20 | 0.58, 2.51 |  | 1.76 | 0.69, 4.53 |
| Standard hours |  | 1.00 | - |  | 1.00 | - |
| Slightly long hours |  | 0.85 | 0.64, 1.14 |  | 0.53 | 0.22, 1.29 |
| Long or very long hours | | 0.90 | 0.63, 1.31 |  | **3.15** | **1.31, 7.61** |
|  | **Smoking** |  |  |  |  |  |
|  | Occasional vs. never | |  |  |  |  |
| Part-time |  | 0.56 | 0.17, 1.87 |  | 1.08 | 0.60, 1.95 |
| Standard hours |  | 1.00 | - |  | 1.00 | - |
| Slightly long hours |  | 1.10 | 0.76, 1.60 |  | **0.61** | **0.39, 0.95** |
| Long or very long hours | | 1.23 | 0.80, 1.90 |  | 1.47 | 0.74, 2.93 |
|  | Former vs. never | |  |  |  |  |
| Part-time |  | 0.98 | 0.61, 1.58 |  | 0.80 | 0.58, 1.10 |
| Standard hours |  | 1.00 | - |  | 1.00 | - |
| Slightly long hours |  | 1.05 | 0.88, 1.26 |  | 1.01 | 0.84, 1.22 |
| Long or very long hours | | 0.95 | 0.76, 1.20 |  | 1.16 | 0.80, 1.68 |
|  | Current light vs. never | |  |  |  |  |
| Part-time |  | 0.62 | 0.24, 1.61 |  | 0.72 | 0.44, 1.16 |
| Standard hours |  | 1.00 | - |  | 1.00 | - |
| Slightly long hours |  | 0.88 | 0.63, 1.24 |  | 1.23 | 0.96, 1.57 |
| Long or very long hours | | 0.83 | 0.54, 1.28 |  | 1.10 | 0.64, 1.88 |
|  | Current medium vs. never | | |  |  |  |
| Part-time |  | 0.80 | 0.37, 1.71 |  | 0.93 | 0.60, 1.45 |
| Standard hours |  | 1.00 | **-** |  | 1.00 | - |
| Slightly long hours |  | **1.37** | **1.07, 1.77** |  | **1.68** | **1.34, 2.10** |
| Long or very long hours | | 1.11 | 0.81, 1.53 |  | **2.23** | **1.45, 3.42** |
|  | Current heavy vs. never | | | | | |
| Part-time |  | 0.73 | 0.36, 1.44 |  | 0.46 | 0.21, 1.03 |
| Standard hours |  | 1.00 | **-** |  | 1.00 | - |
| Slightly long hours |  | 1.10 | 0.87, 1.38 |  | **1.45** | **1.04, 2.03** |
| Long or very long hours | | **1.43** | **1.09, 1.88** |  | **3.53** | **2.18, 5.72** |
|  | **Sleep length** |  |  |  |  |  |
|  | Less than 7h vs. 7-8 hour | | |  |  |  |
| Part-time |  | 1.05 | 0.62, 1.79 |  | 0.89 | 0.63, 1.26 |
| Standard hours |  | 1.00 | **-** |  | 1.00 | - |
| Slightly long hours |  | **1.25** | **1.03, 1.52** |  | **1.40** | **1.17, 1.68** |
| Long or very long hours | | **1.64** | **1.31, 2.04** |  | **1.45** | **1.04, 2.03** |
|  | 8.5 h or more vs. 7-8 hours | | |  |  |  |
| Part-time |  | 1.45 | 0.83, 2.51 |  | **1.61** | **1.20, 2.16** |
| Standard hours |  | 1.00 | - |  | 1.00 | - |
| Slightly long hours |  | **0.77** | **0.61, 0.99** |  | **0.79** | **0.65, 0.98** |
| Long or very long hours | | 0.84 | 0.61, 1.14 |  | 0.69 | 0.44, 1.08 |
|  | **Sleep quality** |  |  |  |  |  |
|  | Poor vs. good | |  |  |  |  |
| Part-time |  | **2.22** | **1.11, 4.44** |  | 1.23 | 0.75, 2.02 |
| Standard hours |  | 1.00 | **-** |  | 1.00 | - |
| Slightly long hours |  | 1.19 | 0.85, 1.65 |  | **1.58** | **1.19, 2.11** |
| Long or very long hours | | 0.95 | 0.62, 1.44 |  | 1.30 | 0.68, 2.50 |
|  | Fairly poor vs. good | |  |  |  |  |
| Part-time |  | 0.84 | 0.44, 1.63 |  | 0.82 | 0.56, 1.20 |
| Standard hours |  | 1.00 | - |  | 1.00 | - |
| Slightly long hours |  | 0.95 | 0.75, 1.2 |  | 1.09 | 0.89, 1.35 |
| Long or very long hours | | 0.91 | 0.68, 1.21 |  | 1.22 | 0.82, 1.80 |
| CI, confidence interval; OR, odds ratio; Ref., reference category. The model was adjusted for baseline age, sex, socioeconomic status, and work schedule. | | | | | | |
| ^a^ Categories long hours and very long hours were combined.  ^b^ CIs were corrected for clustered sampling within families.  ^c^ Categories high and very high were combined. | | | |  |  |  |
| Statistically significant associations are presented in bold. | | | |  |  |  |

**Supplementary Table S8** Association of working hours with all-cause and cardiovascular disease (CVD) mortality at between-pair and within-pair level in all pairs (n=2,874 pairs), in monozygotic (MZ) pairs (1,000 pairs) and in dizygotic (DZ) pairs (1,769 pairs).

|  | Working hours (standard hours as ref.) | HR | 95% CI | HR | 95% CI | HR | 95% CI |
| --- | --- | --- | --- | --- | --- | --- | --- |
|  | |  |  |  |  |  |  |
| **All-cause mortality** | | **All twin pairs** | | **DZ twin pairs** | | **MZ twin pairs** | |
| Between-pair | |  |  |  |  |  |  |
|  | Part-time | **1.52** | **1.03, 2.24** | **2.06** | **1.26, 3.36** | 0.97 | 0.50, 1.87 |
|  | Standard hours | 1.00 | - | 1.00 | - | 1.00 | - |
|  | Slightly long hours | 1.08 | 0.89, 1.31 | **1.37** | **1.07, 1.74** | 0.73 | 0.52, 1.02 |
|  | Long hours | **0.51** | **0.36, 0.73** | **0.57** | **0.35, 0.92** | **0.44** | **0.26, 0.76** |
|  | Very long hours | 0.91 | 0.60, 1.38 | 0.96 | 0.57, 1.61 | 0.69 | 0.34, 1.40 |
| Within-pair | |  |  |  |  |  |  |
|  | Part-time | 1.05 | 0.75, 1.48 | 1.04 | 0.69, 1.58 | 0.89 | 0.48, 1.62 |
|  | Standard hours | 1.00 | - | 1.00 | - | 1.00 | - |
|  | Slightly long hours | 1.00 | 0.83, 1.19 | 0.96 | 0.76, 1.21 | 1.10 | 0.81, 1.49 |
|  | Long hours | 1.01 | 0.73, 1.41 | 0.94 | 0.61, 1.44 | 1.21 | 0.72, 2.06 |
|  | Very long hours | 1.35 | 0.88, 2.07 | 1.21 | 0.69, 2.11 | 1.93 | 0.92, 4.03 |
|  | | |  |  |  |  |  |
| **CVD mortality** | | **All twin pairs** | | **DZ twin pairs** | | **MZ twin pairs** | |
| Between-pair | |  | |  | |  | |
|  | Part-time | 1.55 | 0.74, 3.24 | **2.56** | **1.06, 6.19** | 0.75 | 0.19, 3.00 |
|  | Standard hours | 1.00 | - | 1.00 | - | 1.00 | - |
|  | Slightly long hours | 1.14 | 0.79, 1.64 | 1.52 | 0.96, 2.40 | 0.73 | 0.38, 1.40 |
|  | Long hours | 0.70 | 0.37, 1.32 | 0.41 | 0.15, 1.09 | 1.07 | 0.47, 2.42 |
|  | Very long hours | 1.44 | 0.76, 2.71 | 1.34 | 0.56, 3.18 | 1.56 | 0.61, 3.98 |
| Within-pair | |  |  |  |  |  |  |
|  | Part-time | 1.04 | 0.46, 2.33 | 1.08 | 0.44, 2.67 | 0.85 | 0.14, 5.28 |
|  | Standard hours | 1.00 | - | 1.00 | - | 1.00 | - |
|  | Slightly long hours | 1.22 | 0.86, 1.73 | 1.16 | 0.73, 1.85 | 1.45 | 0.84, 2.52 |
|  | Long hours | 1.68 | 0.96, 2.95 | 1.63 | 0.65, 4.06 | 1.93 | 0.91, 4.09 |
|  | Very long hours | 1.20 | 0.53, 2.73 | 0.81 | 0.27, 2.44 | 2.32 | 0.65, 8.21 |
| CI, confidence interval; HR, hazard ratio estimated from Cox model; Ref., reference category. Model was adjusted for baseline age and sex. Statistically significant associations are presented in bold. | | | | | | | |

**Supplementary Table S9** Association of working hours with biological aging measured by AAPC-Grim and DunedinPACE at between-pair and within-pair level in all pairs (278 pairs), in monozygotic (MZ) pairs (168 pairs) and in dizygotic (DZ) pairs (110 pairs).

|  | Working hours  (standard hours as ref.) | *b* | 95% CI | *b* | 95% CI | *b* | 95% CI |
| --- | --- | --- | --- | --- | --- | --- | --- |
| **AAPC-Grim** | | **All twin pairs** | | **DZ twin pairs** | | **MZ twin pairs** | |
| Between-pair | |  |  |  |  |  |  |
|  | Part-time | **-2.20** | **-4.38, -0.02** | -1.69 | -5.16, 1.78 | -1.96 | -4.76, 0.84 |
|  | Standard hours | 0.00 | - | 0.00 | - | 0.00 | - |
|  | Slightly long hours | 0.42 | -0.79, 1.63 | 1.30 | -0.60, 3.20 | 0.21 | -1.38, 1.80 |
|  | Long or very long hours | 0.53 | -1.29, 2.35 | 1.36 | -2.63, 5.35 | 0.23 | -1.78, 2.24 |
| Within-pair | |  |  |  |  |  |  |
|  | Part-time | -0.53 | -1.81, 0.75 | -1.86 | -4.06, 0.34 | 0.52 | -1.00, 2.04 |
|  | Standard hours | 0.00 | - | 0.00 | - | 0.00 | - |
|  | Slightly long hours | -0.20 | -0.95, 0.55 | -0.17 | -1.57, 1.23 | -0.26 | -1.11, 0.59 |
|  | Long or very long hours | -0.15 | -1.32, 1.02 | -0.48 | -2.75, 1.79 | -0.01 | -1.29, 1.27 |
| **DunedinPACE** | | **All twin pairs** | | **DZ twin pairs** | | **MZ twin pairs** | |
| Between-pair | |  |  |  |  |  |  |
|  | Part-time | -0.06 | -0.13, 0.01 | -0.06 | -0.16, 0.04 | -0.06 | -0.15, 0.03 |
|  | Standard hours | 0.00 | - | 0.00 | - | 0.00 | - |
|  | Slightly long hours | 0.01 | -0.03, 0.05 | 0.03 | -0.02, 0.08 | 0.00 | -0.05, 0.05 |
|  | Long or very long hours | 0.02 | -0.04, 0.08 | 0.06 | -0.05, 0.17 | 0.01 | -0.06, 0.08 |
| Within-pair | |  |  |  |  |  |  |
|  | Part-time | 0.01 | -0.03, 0.05 | 0.01 | -0.06, 0.08 | 0.02 | -0.03, 0.07 |
|  | Standard hours | 0.00 | - | 0.00 | - | 0.00 | - |
|  | Slightly long hours | 0.02 | 0.00, 0.04 | 0.04 | -0.01, 0.09 | 0.00 | -0.03, 0.03 |
|  | Long or very long hours | 0.01 | -0.03, 0.05 | 0.00 | -0.07, 0.07 | 0.01 | -0.03, 0.05 |
| b, unstandardized regression coefficient; CI, confidence interval; Ref., reference category. Model was adjusted for baseline age, sex, and timing of the blood drawn;  Statistically significant associations are presented in bold. | | | | | | | |

**Supplementary Table S10** Association of working hours with continuous lifestyle factors at between-pair and within-pair level in all pairs (2,874 pairs), in monozygotic (MZ) pairs (1,000 pairs) and in dizygotic (DZ) pairs (1,769 pairs).

|  | Working hours (standard hours as ref.) | *b* | *95% CI* | *b* | *95% CI* | *b* | *95% CI* |
| --- | --- | --- | --- | --- | --- | --- | --- |
| **BMI** | | **All twin pairs** | | **DZ twin pairs** | | **MZ twin pairs** | |
| Between-pair | |  |  |  |  |  |  |
|  | Part-time | **-1.28** | **-1.91, -0.65** | **-1.16** | **-1.94, -0.38** | **-1.23** | **-2.35, -0.11** |
|  | Standard hours | 0.00 | - | 0.00 | - | 0.00 | - |
|  | Slightly long hours | **0.39** | **0.08, 0.70** | 0.29 | -0.08, 0.66 | 0.36 | -0.20, 0.92 |
|  | Long hours | 0.25 | -0.28, 0.78 | -0.38 | -1.05, 0.29 | **1.43** | **0.52, 2.34** |
|  | Very long hours | 0.60 | -0.09, 1.29 | 0.14 | -0.71, 0.99 | 0.78 | -0.49, 2.05 |
| Within-pair | |  |  |  |  |  |  |
|  | Part-time | -0.08 | -0.50, 0.34 | -0.17 | -0.78, 0.44 | 0.12 | -0.44, 0.68 |
|  | Standard hours | 0.00 | - | 0.00 | - | 0.00 | - |
|  | Slightly long hours | -0.10 | -0.31, 0.11 | 0.01 | -0.29, 0.31 | -0.26 | -0.54, 0.02 |
|  | Long hours | 0.32 | -0.04, 0.68 | **0.51** | **0.00, 1.02** | -0.03 | -0.50, 0.44 |
|  | Very long hours | **0.56** | **0.07, 1.05** | 0.48 | -0.18, 1.14 | 0.48 | -0.21, 1.17 |
| **LTPA** | | **All twin pairs** | | **DZ twin pairs** | | **MZ twin pairs** | |
| Between-pair | |  |  |  |  |  |  |
|  | Part-time | 0.39 | -0.22, 1.00 | **0.80** | **0.01, 1.59** | -0.21 | -1.22, 0.80 |
|  | Standard hours | 0.00 | - | 0.00 | - | 0.00 | - |
|  | Slightly long hours | -0.17 | -0.47, 0.13 | -0.26 | -0.64, 0.12 | 0.05 | -0.45, 0.55 |
|  | Long hours | -0.30 | -0.81, 0.21 | -0.55 | -1.22, 0.12 | -0.29 | -1.10, 0.52 |
|  | Very long hours | **-1.12** | **-1.79, -0.45** | **-1.08** | **-1.95, -0.21** | -0.99 | -2.13, 0.15 |
| Within-pair | |  |  |  |  |  |  |
|  | Part-time | 0.27 | -0.28, 0.82 | 0.30 | -0.41, 1.01 | 0.36 | -0.50, 1.22 |
|  | Standard hours | 0.00 | - | 0.00 | - | 0.00 | - |
|  | Slightly long hours | -0.06 | -0.34, 0.22 | -0.14 | -0.50, 0.22 | 0.06 | -0.37, 0.49 |
|  | Long hours | -0.43 | -0.89, 0.03 | **-0.73** | **-1.34, -0.12** | -0.42 | -1.13, 0.29 |
|  | Very long hours | -0.54 | -1.17, 0.09 | -0.76 | -1.54, 0.02 | -0.13 | -1.19, 0.93 |
| b, unstandardized regression coefficient; CI, confidence interval; Ref., reference category. Model was adjusted for baseline age and sex. Statistically significant associations are presented in bold. | | | | | | | |

**Supplementary Table S11** Association of working hours with categorical lifestyle factors at between-pair and within-pair level in all twin pairs (2,874 pairs), in monozygotic (MZ) pairs (1,000 pairs) and in dizygotic (DZ) pairs (1,769 pairs).

|  | Working hours  (standard hours as ref.) | OR | 95% CI | OR | 95% CI | OR | 95% CI |
| --- | --- | --- | --- | --- | --- | --- | --- |
|  |  | **All twin pairs** | | **DZ twin pairs** | | **MZ twin pairs** | |
| **Smoking** | | **Occasional vs. never** ^a^ | | | | | |
| Between-pair | |  |  |  |  |  |  |
|  | Part-time | 1.32 | 0.55, 3.16 | 1.11 | 0.33, 3.72 | 0.93 | 0.42, 2.06 |
|  | Standard hours | 1.00 | - | 1.00 | - | 1.00 | - |
|  | Slightly long hours | 0.96 | 0.60, 1.54 | 0.95 | 0.51, 1.76 | 0.99 | 0.67, 1.46 |
|  | Long hours | 1.14 | 0.54, 2.42 | 1.27 | 0.45, 3.61 | 0.65 | 0.34, 1.24 |
|  | Very long hours | 1.25 | 0.47, 3.35 | 1.99 | 0.59, 6.77 | 0.57 | 0.22, 1.48 |
| Within-pair | |  |  |  |  |  |  |
|  | Part-time | 0.71 | 0.28, 1.79 | 2.23 | 0.64, 7.74 | 0.64 | 0.29, 1.43 |
|  | Standard hours | 1.00 | - | 1.00 | - | 1.00 | - |
|  | Slightly long hours | 0.97 | 0.58, 1.61 | 1.07 | 0.55, 2.07 | 0.87 | 0.59, 1.29 |
|  | Long hours | 1.16 | 0.51, 2.61 | 1.66 | 0.57, 4.86 | 1.45 | 0.74, 2.81 |
|  | Very long hours | 0.89 | 0.3, 2.64 | 1.75 | 0.50, 6.20 | 0.71 | 0.26, 1.96 |
|  |  | **Former vs. never** ^a^ | | | | | |
| Between-pair | |  |  |  |  |  |  |
|  | Part-time | 0.61 | 0.36, 1.03 | 0.47 | 0.24, 0.94 | 0.93 | 0.42, 2.06 |
|  | Standard hours | 1.00 | - | 1.00 | - | 1.00 | - |
|  | Slightly long hours | 1.02 | 0.80, 1.29 | 1.09 | 0.80, 1.47 | 0.99 | 0.67, 1.46 |
|  | Long hours | 0.94 | 0.63, 1.41 | 1.25 | 0.74, 2.13 | 0.65 | 0.34, 1.24 |
|  | Very long hours | 0.66 | 0.38, 1.17 | 0.82 | 0.40, 1.67 | 0.57 | 0.22, 1.48 |
| Within-pair | |  |  |  |  |  |  |
|  | Part-time | 1.11 | 0.68, 1.81 | 1.25 | 0.67, 2.34 | 0.64 | 0.29, 1.43 |
|  | Standard hours | 1.00 | - | 1.00 | - | 1.00 | - |
|  | Slightly long hours | 0.87 | 0.69, 1.10 | 0.88 | 0.66, 1.18 | 0.87 | 0.59, 1.29 |
|  | Long hours | 1.10 | 0.74, 1.62 | 1.00 | 0.62, 1.63 | 1.45 | 0.74, 2.81 |
|  | Very long hours | 0.92 | 0.53, 1.60 | 0.87 | 0.46, 1.67 | 0.71 | 0.26, 1.96 |
|  |  | **Current light vs. never** | | | | | |
| Between-pair | |  |  |  |  |  |  |
|  | Part-time | 0.48 | 0.23, 1.03 | 0.60 | 0.24, 1.53 | 0.50 | 0.13, 1.9 |
|  | Standard hours | 1.00 | - | 1.00 | - | 1.00 | - |
|  | Slightly long hours | 0.88 | 0.62, 1.24 | 0.94 | 0.61, 1.46 | 0.84 | 0.46, 1.56 |
|  | Long hours | 0.59 | 0.30, 1.16 | 0.37 | 0.14, 0.98 | 1.17 | 0.45, 3.05 |
|  | Very long hours | 0.60 | 0.24, 1.52 | 0.39 | 0.11, 1.39 | 1.10 | 0.24, 4.94 |
| Within-pair | |  |  |  |  |  |  |
|  | Part-time | 0.71 | 0.33, 1.53 | 0.69 | 0.27, 1.74 | 0.78 | 0.18, 3.34 |
|  | Standard hours | 1.00 | - | 1.00 | - | 1.00 | - |
|  | Slightly long hours | 1.1 | 0.76, 1.60 | 1.27 | 0.81, 2.02 | 0.74 | 0.37, 1.45 |
|  | Long hours | 0.95 | 0.47, 1.92 | 0.89 | 0.33, 2.38 | 1.01 | 0.33, 3.11 |
|  | Very long hours | 1.28 | 0.48, 3.42 | 1.20 | 0.34, 4.27 | 1.77 | 0.31, 10.07 |
|  |  | **Current medium vs. never** | | | | | |
| Between-pair | |  |  |  |  |  |  |
|  | Part-time | 0.77 | 0.40, 1.47 | 0.90 | 0.40, 2.03 | 0.82 | 0.27, 2.50 |
|  | Standard hours | 1.00 | - | 1.00 | - | 1.00 | - |
|  | Slightly long hours | **1.55** | **1.15, 2.10** | **1.58** | **1.08, 2.30** | 1.63 | 0.98, 2.73 |
|  | Long hours | 1.13 | 0.68, 1.90 | 1.16 | 0.59, 2.29 | 1.34 | 0.59, 3.06 |
|  | Very long hours | 1.58 | 0.82, 3.03 | 1.27 | 0.54, 3.00 | 2.4 | 0.79, 7.30 |
| Within-pair | |  |  |  |  |  |  |
|  | Part-time | 1.19 | 0.61, 2.31 | 1.14 | 0.51, 2.55 | 1.35 | 0.41, 4.46 |
|  | Standard hours | 1.00 | - | 1.00 | - | 1.00 | - |
|  | Slightly long hours | 1.28 | 0.94, 1.75 | 1.28 | 0.87, 1.89 | 1.16 | 0.67, 2.02 |
|  | Long hours | 1.11 | 0.65, 1.87 | 1.17 | 0.60, 2.27 | 1.03 | 0.41, 2.57 |
|  | Very long hours | 0.84 | 0.43, 1.68 | 1.27 | 0.55, 2.93 | 0.31 | 0.08, 1.16 |
|  |  | **Current heavy vs. never** | | | | | |
| Between-pair | |  |  |  |  |  |  |
|  | Part-time | 0.83 | 0.39, 1.79 | 0.72 | 0.25, 2.06 | 0.97 | 0.29, 3.24 |
|  | Standard hours | 1.00 | - | 1.00 | - | 1.00 | - |
|  | Slightly long hours | **1.54** | **1.11, 2.13** | **1.81** | **1.19, 2.75** | 1.33 | 0.78, 2.27 |
|  | Long hours | 1.58 | 0.96, 2.60 | **2.12** | **1.10, 4.10** | 1.13 | 0.51, 2.50 |
|  | Very long hours | **2.87** | **1.58, 5.22** | **3.33** | **1.52, 7.31** | 2.61 | 0.98, 6.94 |
| Within-pair | |  |  |  |  |  |  |
|  | Part-time | 0.62 | 0.29, 1.34 | 0.56 | 0.20, 1.56 | 0.48 | 0.14, 1.72 |
|  | Standard hours | 1.00 | - | 1.00 | - | 1.00 | - |
|  | Slightly long hours | 0.92 | 0.66, 1.29 | 1.01 | 0.66, 1.54 | 0.88 | 0.49, 1.55 |
|  | Long hours | **1.86** | **1.12, 3.09** | **2.11** | **1.11, 4.03** | **1.58** | 0.66, 3.78 |
|  | Very long hours | 1.45 | 0.78, 2.71 | 1.62 | 0.76, 3.44 | 0.93 | 0.29, 2.97 |
|  |  |  |  |  |  |  |  |
|  |  | **All twin pairs** | | **DZ twin pairs** | | **MZ twin pairs** | |
| **Sleep length** | | **Less than 7h vs. 7-8h** | | | | | |
| Between-pair | |  |  |  |  |  |  |
|  | Part-time | 1.06 | 0.67, 1.67 | 0.89 | 0.49, 1.63 | 1.41 | 0.65, 3.05 |
|  | Standard hours | 1.00 | - | 1.00 | - | 1.00 | - |
|  | Slightly long hours | **1.71** | **1.39, 2.11** | **1.75** | **1.34, 2.28** | **1.7** | **1.18, 2.45** |
|  | Long hours | **1.75** | **1.24, 2.47** | 1.41 | 0.88, 2.25 | **1.96** | **1.12, 3.43** |
|  | Very long hours | **2.66** | **1.73, 4.08** | **2.91** | **1.67, 5.07** | **2.28** | **1.06, 4.9** |
| Within-pair | |  |  |  |  |  |  |
|  | Part-time | 1.01 | 0.62, 1.65 | 1.16 | 0.62, 2.17 | 0.77 | 0.33, 1.83 |
|  | Standard hours | 1.00 | - | 1.00 | - | 1.00 | - |
|  | Slightly long hours | 1.01 | 0.80, 1.28 | 0.97 | 0.72, 1.30 | 1.10 | 0.73, 1.65 |
|  | Long hours | 1.04 | 0.71, 1.51 | 0.96 | 0.58, 1.57 | 0.98 | 0.52, 1.87 |
|  | Very long hours | **1.85** | **1.13, 3.01** | 1.53 | 0.86, 2.75 | **2.60** | **1.02, 6.64** |
|  |  | **8.5h or more vs. 7-8h** | | | | | |
| Between-pair | |  |  |  |  |  |  |
|  | Part-time | 1.24 | 0.77, 1.98 | 0.91 | 0.48, 1.74 | **2.26** | **1.07, 4.76** |
|  | Standard hours | 1.00 | - | 1.00 | - | 1.00 | - |
|  | Slightly long hours | 0.81 | 0.63, 1.05 | 0.84 | 0.61, 1.16 | 0.79 | 0.50, 1.25 |
|  | Long hours | 0.89 | 0.56, 1.40 | 0.82 | 0.45, 1.49 | 0.98 | 0.45, 2.11 |
|  | Very long hours | 0.77 | 0.40, 1.48 | 0.64 | 0.27, 1.51 | 0.97 | 0.30, 3.07 |
| Within-pair | |  |  |  |  |  |  |
|  | Part-time | **1.98** | **1.18, 3.32** | **2.43** | **1.24, 4.79** | 1.22 | 0.52, 2.88 |
|  | Standard hours | 1.00 | - | 1.00 | - | 1.00 | - |
|  | Slightly long hours | 0.78 | 0.58, 1.03 | 0.85 | 0.60, 1.20 | 0.72 | 0.43, 1.22 |
|  | Long hours | 1.28 | 0.78, 2.10 | 1.41 | 0.76, 2.64 | 0.99 | 0.40, 2.44 |
|  | Very long hours | 0.67 | 0.32, 1.37 | 0.62 | 0.25, 1.49 | 0.60 | 0.15, 2.39 |
| CI, confidence interval; OR, odds ratio; Ref., reference category. Model was adjusted for baseline age and sex. Statistically significant associations are presented in bold.  a For MZ pairs, occasional and former were combined. | | | | | | | |

**Supplementary Figure S1** Natural indirect effects of working hours on A) all-cause and B) cardiovascular disease (CVD) mortality on the hazard difference (HD) scale (per 100,000 person-years) through multiple mediators (n = 8550). BMI, body mass index; LTPA, leisure-time physical activity. Standard working hours was treated as the reference category. LTPA and BMI were modeled as continuous, with smoking (never smoked [reference], occasional, former, light, medium, and heavy smoker) and sleep length (7‒8 hours [reference], < 7 hours, and > 8 hours) as categorical. The model was controlled for age, sex, socioeconomic status, and work schedule. The confidence intervals were corrected for non-independence due to family relatedness using a bootstrap approach.

**Supplementary Figure S2** Natural indirect effects (NIEs) and natural direct effects (NDEs) of working hours on biological aging measured by A) AAPC-Grim and B) DunedinPACE (n = 709). Standard working hours was treated as the reference category. Smoking was included as a mediator and modeled as a categorical variable (never smoked [reference category], former/occasional smokers, and current smokers). The model was adjusted for baseline age, sex, timing of the blood drawn, socioeconomic status, and work schedule. The confidence intervals were corrected for non-independence due to family relatedness using a bootstrap approach.

**Supplementary Figure S3** Natural indirect effects of working hours on A) all-cause and B) cardiovascular disease (CVD) mortality in the male participants on the hazard difference (HD) scale (per 100,000 person-years) through one mediator at a time (n = 4192). BMI, body mass index; CI, confidence interval; LTPA, leisure-time physical activity. Standard working hours was treated as the reference category. LTPA and BMI were modeled as continuous, with smoking (never smoked [reference], occasional, former, light, medium, and heavy smoker) and sleep length (7‒8 hours [reference], < 7 hours, and > 8 hours) as categorical. The model was controlled for baseline age, socioeconomic status, and work schedule. The CIs were corrected for non-independence due to family relatedness using a bootstrap approach.

**Supplementary Figure S4** Natural indirect effects of working hours on A) all-cause and B) cardiovascular disease (CVD) mortality in the female participants on the hazard difference (HD) scale (per 100,000 person-years) through one mediator at a time (n = 4358). BMI, body mass index; CI, confidence interval; LTPA, leisure-time physical activity. Standard working hours was treated as the reference category. LTPA and BMI were modeled as continuous, with smoking (never smoked [reference], occasional, former, light, medium, and heavy smoker) and sleep length (7‒8 hours [reference], < 7 hours, and > 8 hours) as categorical. The model was controlled for baseline age, socioeconomic status, and work schedule. The CIs were corrected for non-independence due to family relatedness using a bootstrap approach.
